# The causality between leisure sedentary behaviors, physical activity and obstructive sleep apnea: a bidirectional Mendelian randomization study

**DOI:** 10.3389/fpubh.2024.1425060

**Published:** 2024-06-21

**Authors:** Haonan Tian, Aozhe Wang, Han Wu, Cailiang Zhou, Zhenglong Zhang, Jun Wang

**Affiliations:** ^1^Department of Exercise Physiology, Beijing Sport University, Beijing, China; ^2^Key Laboratory of Physical Fitness and Exercise, Ministry of Education, Beijing Sport University, Beijing, China; ^3^Department of Graduate School, Harbin Sport University, Harbin, China

**Keywords:** physical activity, leisure sedentary behaviors, obstructive sleep apnea, Mendelian randomization, causal relationship

## Abstract

**Background:**

Previous observational studies have shown a correlation between leisure sedentary behaviors (LSB) and physical activity (PA) with the incidence of obstructive sleep apnea (OSA). However, the causal associations remain unknown. Therefore, our study used bidirectional two-sample Mendelian randomization (MR) to identify potential causal relationships between LSB/PA and OSA.

**Methods:**

We sourced genetic variation data for LSB and PA from the UK Biobank, while data on OSA were collected from the FinnGen study. The primary analysis method employed was the inverse variance weighted (IVW) approach, complemented by the weighted median and MR-Egger methods. For sensitivity analyses, we conducted Cochran’s Q test, the MR-Egger intercept test, the MR-PRESSO global test, and the leave-one-out analysis.

**Results:**

IVW analyses showed that genetically predicted leisure television watching (odds ratio [OR] = 1.38, 95% confidence interval [CI] = 1.09–1.75, *p* = 0.007) and computer use (OR = 1.48, 95% CI = 1.15–1.92, *p* = 0.002) significantly increased the risk of OSA. Conversely, self-reported vigorous physical activity (VPA) (OR = 0.33, 95% CI = 0.11–0.98, *p* = 0.046) may reduce the risk of OSA. No causal effects on OSA risk were observed for driving or self-reported moderate-to-vigorous physical activity. Furthermore, the reverse MR analysis indicated no significant causal relationship between OSA and any LSB/PA phenotype. Sensitivity tests showed no significant heterogeneity or horizontal pleiotropy.

**Conclusion:**

This study suggests that leisurely television watching and computer use are risk factors for OSA, while VPA may be a protective factor. Additionally, OSA does not affect PA or LSB levels. We recommend reducing sedentary activities, particularly television watching and computer use, and prioritizing VPA to reduce the risk of OSA. Further research in diverse populations and settings is needed to validate these findings.

## 1 Introduction

Obstructive sleep apnea (OSA) is a sleep disorder characterized by repeated collapse or complete obstruction of the upper airway during sleep, resulting in apnea or hypoventilation. The primary pathophysiological characteristics of OSA include intermittent hypoxia and sleep fragmentation (1). OSA is closely associated with metabolic disorders, cardiovascular diseases, and neuropsychiatric diseases (2–6). OSA has become a significant global public health challenge, affecting approximately one billion adults worldwide (7, 8). Therefore, it is crucial to have a comprehensive understanding of the potential risks and protective factors for OSA to develop new prevention and intervention measures.

Leisure sedentary behaviors (LSB) is defined as low-energy expenditure activities in which the metabolic equivalent is ≤1.5 for maintaining body posture by lying supine or sitting during waking hours, including the three phenotypes of television watching, computer use, and driving (9). Physical activity (PA) is defined as musculoskeletal movement that consumes energy (10). Advances in technology have resulted in significant lifestyle changes, including an increased prevalence of LSB and a reduced level of PA. The coronavirus disease 2019 pandemic has further exacerbated this trend by limiting opportunities for PA while promoting LSB (11). Scientific evidence has shown that LSB is associated with higher all-cause mortality rates, while PA can lower this risk (12).

Observational research consistently demonstrates a positive correlation between LSB and an increased risk of OSA (13–15), while PA is inversely associated with OSA risk (16). The nocturnal movement of fluid from the lower extremities toward the neck is a predisposing factor to OSA (1). LSB impedes venous return, leading to fluid accumulation in the lower extremities (17). In contrast, PA reduces the risk of OSA by activating the muscular venous pump, thereby increasing venous return and reducing fluid accumulation in the lower extremities (18). Additionally, some studies suggest that OSA may lead to lower PA levels (19, 20). OSA-induced hypoxia may lead to the transformation of type I muscle fibers into type II muscle fibers (20). Type I muscle fibers are slow muscle fibers that produce energy through oxidative metabolism and are resistant to fatigue. Type II muscle fibers are fast muscle fibers that produce energy through the glycolytic pathway and are more susceptible to fatigue. The OSA-induced transformation of the type of muscle fiber leads to a decrease in overall PA levels. At the same time, OSA increases inflammatory cytokines expression (21). Inflammatory cytokines not only act directly on muscle fibers, interfering with their signaling and metabolic processes, leading to the accelerated breakdown of muscle proteins, but also affect the function of mitochondria in muscle fibers, thereby affecting muscle contraction efficiency and endurance (22, 23). These changes lead to greater muscle fatigue, further reducing PA levels. These mechanisms illustrate the potential bidirectional causality between LSB, PA, and OSA. On the one hand, increased sedentary behavior and reduced physical activity can exacerbate OSA through fluid redistribution and metabolic changes. On the other hand, OSA can contribute to decreased physical activity through muscle fiber transformation, increased fatigue, and inflammation.

However, it is not possible to establish a causal inference between LSB/PA and OSA in observational studies due to the limitations in evidence quality, the potential for reverse causation, and the influence of residual confounders. Mendelian randomization (MR) offers a powerful alternative to overcome these limitations by using genetic variations as instrumental variables (IVs) to explore causal relationships between exposures and outcomes based on data from Genome-Wide Association Studies (GWAS) (24). This approach capitalizes on the random assortment of genetic variants at conception, ensuring that environmental risk factors do not influence these genetic variants (25). This randomization process effectively reduces the impact of confounding variables and reverse causality, providing a more reliable basis for causal inference compared to traditional observational studies (26).

Although there is a wealth of research on the association between LSB/PA and OSA, there is a scarcity of studies employing MR to disentangle these relationships. Recently, a valuable MR study explored the causal associations between 34 modifiable risk factors and OSA (27). This study found that vigorous PA could reduce the risk of developing OSA, whereas moderate PA and sedentary behavior did not significantly affect OSA. However, this study determined the phenotypes of moderate PA and vigorous PA using the criterion of “days per week with at least 10 min of moderate PA or vigorous PA,” which has certain limitations. Additionally, the study did not classify the LSB phenotypes, despite the possibility that different LSB phenotypes might have varying impacts on OSA risk. Moreover, no MR studies have examined the effects of OSA on LSB and PA. Our study addresses these gaps using a bidirectional two-sample MR method to assess the causal relationship between LSB/PA and OSA. In our study, PA is determined by calculating weekly metabolic equivalent minutes, providing a more accurate measure of activity. We also provide detailed classifications of LSB phenotypes, including television watching, non-work-related computer use, and driving, to explore the specific impacts of different LSB phenotypes on OSA. Additionally, we investigate the effects of OSA on PA and LSB. Our study uncovers more precise causal relationships by addressing previous research limitations and employing a more detailed and accurate classification of PA and LSB. This comprehensive understanding provides valuable insight into potential preventive intervention strategies for OSA and informs better-targeted interventions.

## 2 Methods

### 2.1 Study design

**Figure 1:**
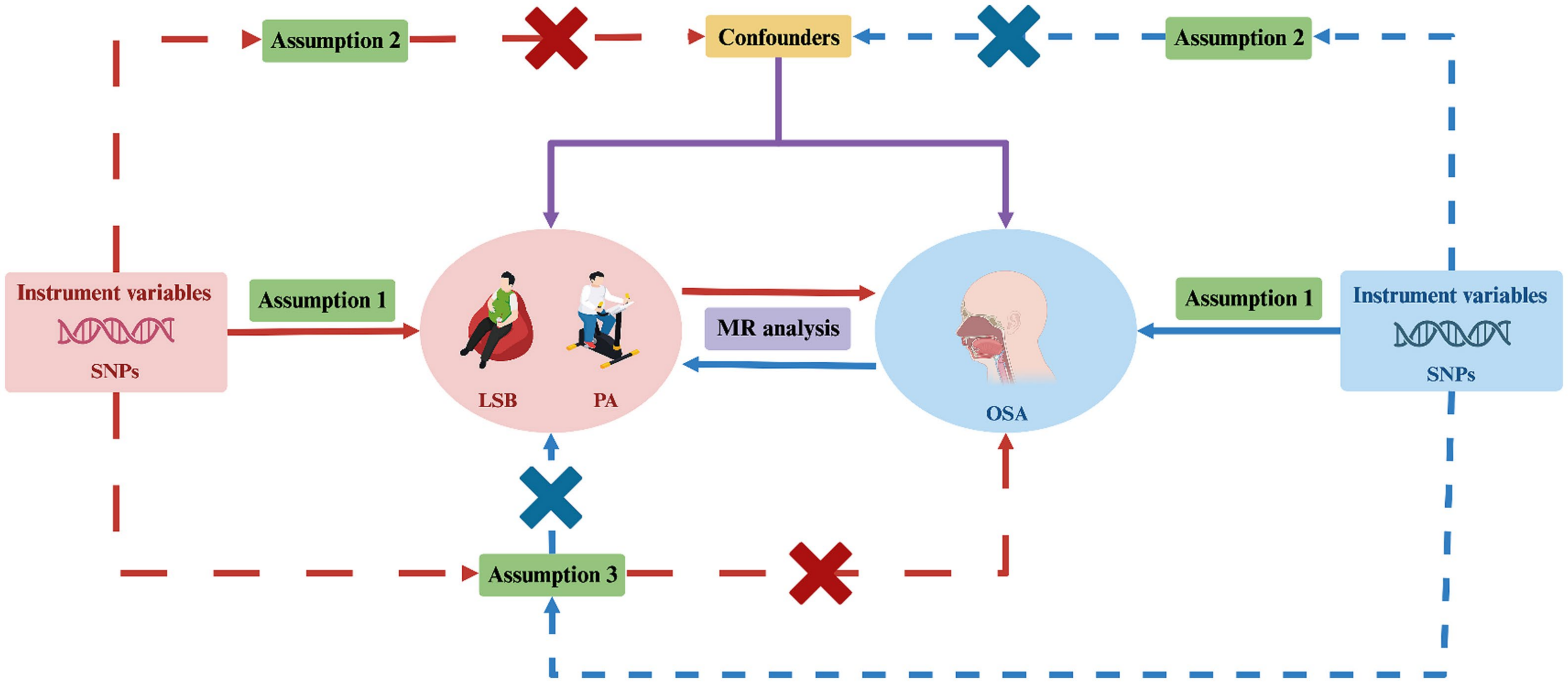
Study design for investigating the causal relationship between LSB/PA and OSA using bidirectional two-sample MR. MR, Mendelian randomization; SNPs, single-nucleotide polymorphisms; LSB, leisure sedentary behavior; PA, physical activity; OSA, obstructive sleep apnea. Created with BioRender.com (agreement number: DY26TQ4FRC).

Our bidirectional two-sample MR analysis consisted of four key steps. First, we identified single nucleotide polymorphisms (SNPs) associated with exposures from large-scale GWAS in publicly available databases. These SNPs served as IVs to ensure the robustness of the MR analyses. Second, we selected genetic data related to outcomes from different databases to prevent sample overlap and potential biases. In the third step, we conducted the bidirectional two-sample MR analysis, using the identified SNPs as instrumental variables to explore the causal relationships in both directions. Finally, we conducted a sensitivity analysis to confirm the robustness of our result. To ensure the reliability of MR findings, three key assumptions must be met: (i) the IVs are closely associated with the specific exposures; (ii) the IVs are independent of any potential confounders; and (iii) the IVs only affect outcomes through exposures (28). Additionally, to avoid biases arising from racial differences, both the genetic data for exposures and outcomes in our MR study were derived from populations of European ancestry. Figure 1 illustrates the design framework for this study.

### 2.2 Data source of LSB/PA

PA related summary statistics were derived from published GWAS studies (Table 1) based on those conducted by the UK Biobank (29). The UK Biobank is a large-scale prospective cohort research program that includes over 500,000 participants of European ancestry, aged between 40 and 69 years, recruited from across the UK. The data collection period spanned from 2006 to 2010, with comprehensive information gathered on various health and lifestyle factors using validated scales (30). This study used two PA phenotypes: self-reported moderate-to-vigorous physical activity (MVPA) and self-reported vigorous physical activity (VPA). Self-reported PA data were collected using a touch-screen questionnaire, a fully validated short version of the International Physical Activity Questionnaire (31–33). The questionnaire evaluated 377,234 participants’ PA levels. For moderate PA, participants were asked: “On how many days in a typical week do you engage in at least 10 min of moderate physical activity, such as light weight-bearing activities or riding at a normal pace? (excluding walking).” For VPA, participants were asked: “On how many days in a typical week do you perform at least 10 min of vigorous physical activity? (These are activities that make you sweat or have difficulty breathing, such as fast cycling, aerobics, and weight lifting).” After removing outliers, MVPA was obtained by calculating the sum of the metabolic equivalents of moderate PA and the metabolic equivalents of VPA. The skewed distribution of the MVPA data necessitated an inverse normalization before conducting the GWAS analysis. VPA has been shown to have high heritability (29), so it was analyzed separately. It is important to note that the VPA data exhibited high skewness and had zero inflation, so it was processed as a dichotomous result to facilitate subsequent analyses.

**Table 1:** MR analyses data sources.

Phenotype	Consortium	Sample size	GWAS ID	Ethnicity
Television watching	UK Biobank (MRC-IEU)	437,887 participants	ukb-b-5192	European
Computer use	UK Biobank (MRC-IEU)	360,895 participants	ukb-b-4522	European
Driving	UK Biobank (MRC-IEU)	310,555 participants	ukb-b-3793	European
MVPA	UK Biobank	377,234 participants	ebi-a-GCST006097	European
VPA	UK Biobank	377,234 participants	ebi-a-GCST006098	European
OSA	FinnGen Biobank	16,761 patients/201,194 controls	finn-b-G6_SLEEPAPNO	European

OSA, obstructive sleep apnea; MVPA, self-reported moderate-to-vigorous physical activity; VPA, self-reported vigorous physical activity.

Data related to LSB are derived from the MRC IEU OpenGWAS database,^1^ developed by the MRC Integrated Epidemiology Unit at the University of Bristol (34). The database currently contains 346.6 billion genetic associations from 50,044 GWAS datasets covering different human phenotypes and disease outcomes in different populations. The LSB consists of three phenotypes: television watching, non-work-related computer use, and driving (Table 1). All three phenotypes were obtained from the UK Biobank data section of the MRC IEU OpenGWAS database (35). To assess the LSB, each participant was asked three questions: “How many hours do you spend watching television each day?,” “How many hours do you spend using a computer for non-work purposes each day?,” and “How many hours do you spend driving each day?.” Based on these questions, the number of individuals exhibiting phenotypes of television watching, non-work-related computer use, and driving is 437,887, 360,895, and 310,555, respectively.

### 2.3 Data source of OSA

We extracted candidate genetic instruments for OSA from a recent GWAS involving 16,761 patients and 201,194 controls in the FinnGen Study (36). FinnGen^2^ is a large-scale biobank project in Finland that aims to collect and analyze genomic and health data from 500,000 participants to improve human health through genetic research. OSA diagnoses were based on the International Statistical Classification of Diseases (ICD) codes (ICD-10: G47.3, R06.5; ICD-9:3472A), and were supplemented by clinical examination, subjective symptoms, and sleep registration with an apnoea-hypopnoea index ≥5/h or a respiratory event index ≥5/h. The FinnGen dataset consisted of individuals of European ancestry.

### 2.4 Selection of instrumental variables

**Figure 2:**
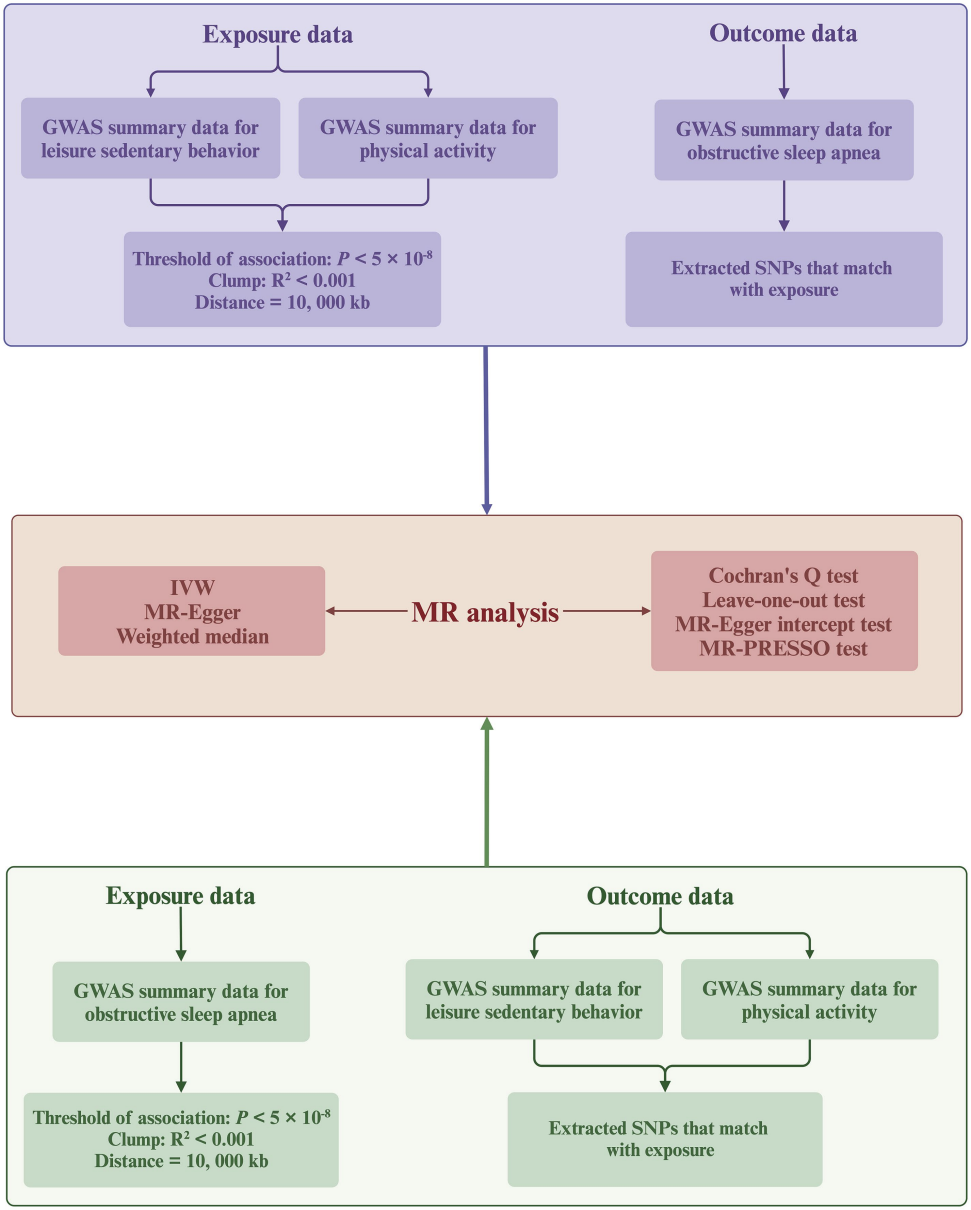
Flowchart of the bidirectional MR analysis. MR, Mendelian randomization; SNPs, single-nucleotide polymorphisms; IVW, inverse-variance weighted; GWAS, genome-wide association study. Created with BioRender.com (agreement number: KT26TQ67AD).

We implemented a meticulous selection process to ensure the quality of IVs, aiming to meet the three core assumptions required for MR analysis (Figure 2). Initially, we identified genome-wide significant SNPs from the GWAS of the targeted exposure (*p* < 5 × 10^−8^). Second, the PLINK algorithm was used to identify SNPs not in linkage disequilibrium (*r*^2^ < 0.001; clumping distance = 10,000 kb). Third, we manually screened and excluded any IVs potentially related to the outcome traits using the PhenoScanner V2 database.^3^ Finally, we excluded SNPs with an F-statistic <10 to prevent biases caused by weak instruments. The F-statistic is calculated using the formula $F=\frac{R^{2}\times \left(N-2\right)}{1-R^{2}}$ , where *N* denotes the sample size and *R^2^* represents the proportion of the variance in the exposure that is explained by the IVs (37, 38).

### 2.5 Mendelian randomization analyses

This study utilizes a bidirectional two-sample MR approach (Figure 2), primarily employing the inverse variance weighted (IVW) method to deduce causal relationships. The IVW method utilizes meta-analysis to aggregate Wald ratio estimates from each SNP, deriving a consolidated effect estimate (39). Under the assumption that all SNPs are valid IVs (0% null IVs), meaning there is no horizontal pleiotropy (IVs influence the outcome only through the exposure), the IVW method is considered the most reliable for causal inference among all MR analysis approaches (40). However, the IVW method may lead to biased conclusions in the presence of horizontal pleiotropy (41). Therefore, we supplement the IVW method with the weighted median and MR-Egger methods. The weighted median method can provide consistent causal estimates even if up to 50% of the SNPs violate the IVs validity assumption (50% null IVs) (42). The MR-Egger method provides robust estimates under the scenario where all SNPs violate the IVs validity assumption (100% null IVs) (43). In cases of inconsistency across the directional results of different MR analyses, we establish a stricter instrument *p*-value threshold to ensure reliability (44).

### 2.6 Sensitivity analyses

To ensure the robustness and reliability of the MR results, we performed various sensitivity analyses. Initially, Cochran’s Q test assessed heterogeneity among SNPs in the IVW analysis (45). The *p*-value > 0.05 indicates no significant heterogeneity, allowing the use of a fixed effect model. We employed the IVW method with a multiplicative random effects model in the presence of heterogeneity (*p*-value < 0.05). Subsequently, we applied the Mendelian randomization pleiotropy residual sum and outlier (MR-PRESSO) test to identify SNPs outliers with pleiotropic effects. After removing outliers, we performed an outlier-adjusted MR analysis to produce unbiased causal effect estimates (46). Third, the MR-Egger regression intercept test was used to evaluate horizontal pleiotropy. MR-Egger regression, developed from Egger regression, uses the formula $\alpha_{i}=\beta_{\gamma i}+\beta_{0}$ , where $\alpha_{i}$ represents the effect of the IVs on the outcome, $\beta_{\gamma i}$ represents the indirect effect of the IVs on the outcome through the exposure, and $\beta_{0}$ represents the estimated average horizontal pleiotropic effect. An intercept $\beta_{0}$ with *p*-value < 0.05 indicates the presence of horizontal pleiotropy (43). Finally, we conducted a leave-one-out (LOO) analysis to investigate whether specific SNPs strongly drive the causal relationship. This method sequentially excluded SNPs associated with the exposure and repeated the IVW analysis to observe if there were statistical differences in the results before and after exclusion.

### 2.7 Statistical analysis

All statistical analyses were conducted using the TwoSampleMR (version 0.5.7) and MR-PRESSO (version 1.0) packages within R (version 4.3.3). In this study, due to the multiple tests involving five exposure variables, a Bonferroni-adjusted *p*-value < 0.01 was used as the significance threshold to rigorously control the overall Type I error rate. Associations with a *p*-value below 0.05 but above 0.01 were regarded as nominal evidence of association, indicating potential relationships that may merit further investigation. The results of the MR analyses are presented as odds ratios (OR) with the corresponding 95% confidence interval (CI).

### 2.8 Ethics statement

All data used in this study were derived from publicly accessible online GWAS databases. The original authors of these GWAS had already secured all necessary ethical approvals and participant consents. Because our research does not involve direct interaction with human subjects or the collection of new data, but rather re-analyzes existing datasets, it does not require additional ethical or moral review.

## 3 Results

We applied strict selection criteria to identify 187 SNPs associated with LSB, which were subsequently used as IVs. Of these SNPs, 106 were associated with television watching, 76 with computer use, and 5 with driving. All F-statistics used for the IVs of LSB were > 10, ranging from 16.60 to 83.85. The median F-statistics for television watching, computer use, and driving were 20.61, 21.43, and 17.69, respectively, suggesting that there is no weak instrument bias. Supplementary Tables S1–S3 present detailed data. For PA, a total of 24 independent SNPs were selected as IVs. Of these, 17 SNPs were associated with MVPA and 7 SNPs with VPA. All F-statistics for the IVs used for PA exceeded 10, ranging from 27.51 to 58.64. The median F-statistics for MVPA and VPA were 29.39 and 39.67, respectively, suggesting that the weak instrumental bias was absent. Supplementary Tables S4, S5 list the results. The reverse MR analysis included a total of 5 independent SNPs as IVs of OSA. All F-statistics used for the IV of OSA were > 10, ranging from 504.94 to 1117.14, with a median of 542.04. This suggests that the weak instrumental bias is not present. Supplementary Table S6 presents the results.

### 3.1 Causal effect of LSB/PA on OSA

**Figure 3:**
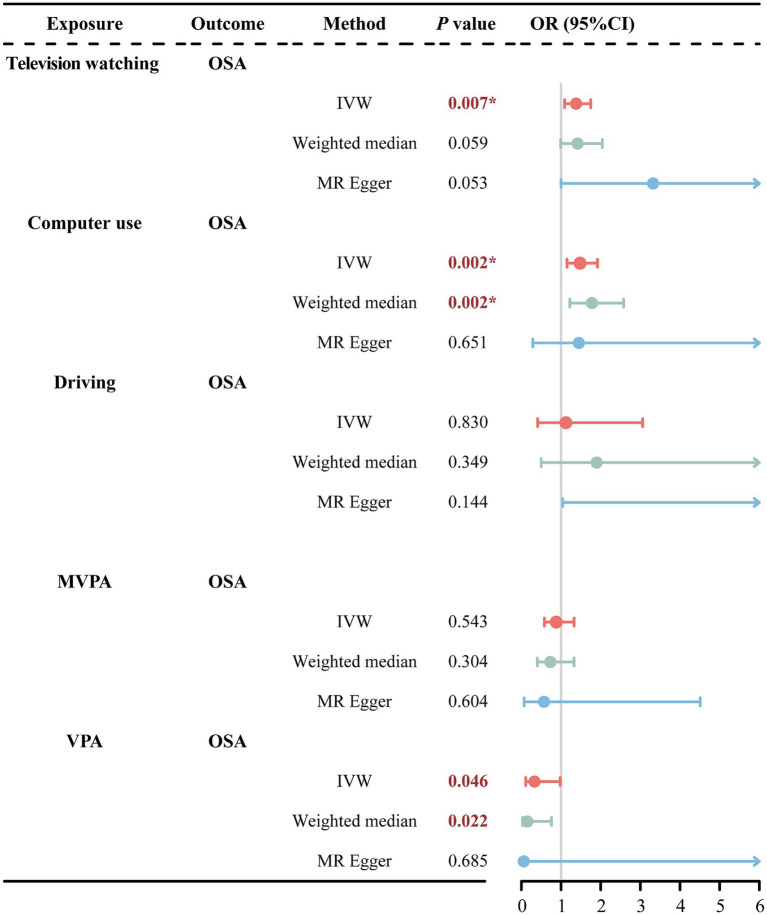
MR estimates results of causal effect of LSB/PA on OSA. OSA, obstructive sleep apnea; MVPA, self-reported moderate-to-vigorous physical activity; VPA, self-reported vigorous physical activity; IVW, inverse variance weighted; OR, odds ratios; 95%CI, 95% confidence interval; *p < 0.01.

IVW analysis revealed that television watching significantly increased the risk of OSA (OR = 1.38, 95% CI = 1.09–1.75, *p* = 0.007) as shown in Figure 3. In addition, the weighted median and MR-Egger methods showed a consistent direction, although the observed trends were not statistically significant. For the causality between computer use and OSA, IVW analysis revealed a significant association between computer use and increased risk of OSA (OR = 1.48, 95% CI = 1.15–1.92, *p* = 0.002). Similarly, weighted median analysis supported this causal association (OR = 1.78, 95% CI = 1.22–2.58, *p* = 0.002). The MR-Egger method analysis showed a consistent direction, although the observed trends were not statistically significant. However, among these three methods of MR analysis, there was no evidence to suggest a causal association between driving and OSA.

In the PA phenotype, IVW analysis showed a nominal association between VPA and reduced risk of OSA (OR = 0.33, 95% CI = 0.11–0.98, *p* = 0.046). The weighted median analysis also presented the nominal association (OR = 0.15, 95% CI = 0.03–0.76, *p* = 0.022). Consistently, the MR-Egger method showed a similar direction, though not statistically significant. However, among these three methods of MR analysis—IVW, Weighted Median, and MR-Egger—there was no statistically significant evidence (*p* > 0.05) to suggest a causal association between driving and OSA.

### 3.2 Causal effect of OSA on LSB/PA

**Figure 4:**
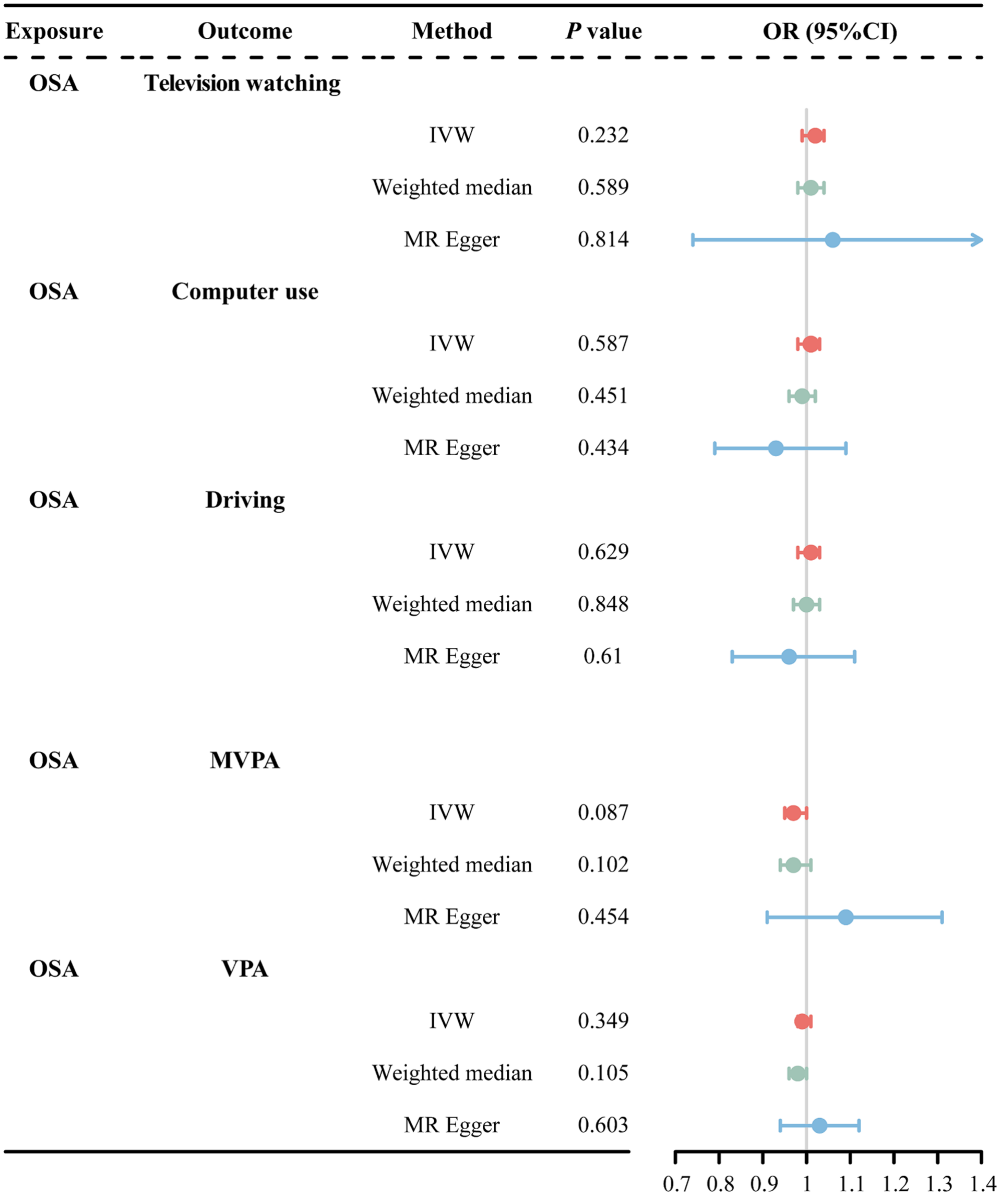
MR estimates results of causal effect of OSA on LSB/PA. OSA, obstructive sleep apnea; MVPA, self-reported moderate-to-vigorous physical activity; VPA, self-reported vigorous physical activity; IVW, inverse variance weighted; OR, odds ratios; 95%CI, 95% confidence interval.

IVW analysis revealed a nominally significant association between OSA and MVPA (OR = 0.97, 95% CI = 0.94–0.99, *p* = 0.048). However, the MR-Egger method showed inconsistent directions (Supplementary Figure S1). Therefore, we used a more stringent genome-wide significance level (*p* < 3 × 10^−8^) while keeping the rest of the screening conditions unchanged. Re-performing the MR analysis after excluding rs142006783 (*p* = 4.8 × 10^−8^) showed that the previously observed significant association between OSA and MVPA disappeared (Figure 4). Additionally, the weighted median and MR-Egger methods remained in a consistent direction. This suggests that there is no causal association between OSA and MVPA. Furthermore, our analyses failed to reveal that OSA causally affects changes in TV watching, computer use, and VPA (Figure 4).

### 3.3 Sensitivity analyses

**Figure 5:**
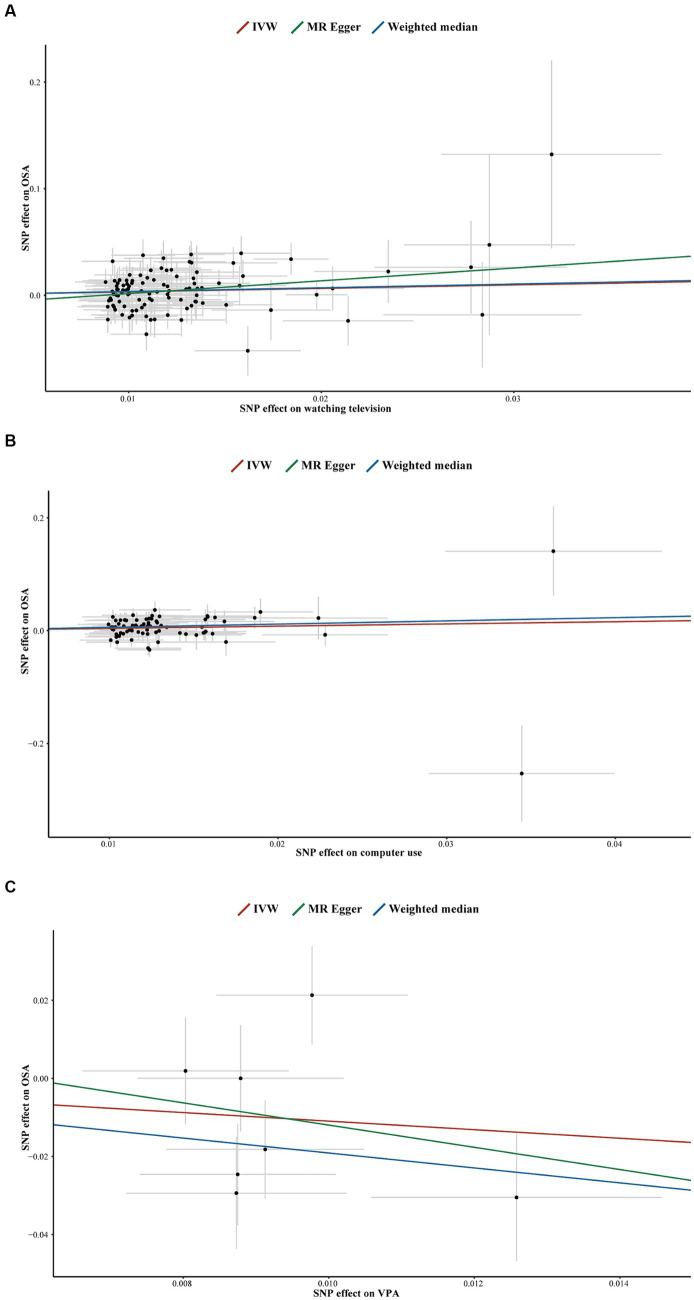
Scatter plots for television watching on OSA **(A)**, computer use on OSA **(B)**, VPA on OSA **(C)**. SNP, single-nucleotide polymorphisms; OSA, obstructive sleep apnea; IVW, inverse variance weighted; VPA, self-reported vigorous physical activity.

In the forward MR analyses, all *p*-values from Cochran’s Q test were > 0.05, indicating no significant heterogeneity (Table 2). Similarly, no horizontal pleiotropy was detected between LSB/PA and OSA, as indicated by *p*-values > 0.05 for both the MR-Egger intercept test (Figure 5; Supplementary Figures S2, S3) and the MR-PRESSO global test (Table 2). Furthermore, the LOO analyses showed that no single SNP significantly influenced the results (Supplementary Figures S4–S8).

**Table 2:** Sensitivity analysis of the causal association between LSB, PA and the risk of OSA.

Exposure	Outcome	Cochran’s Q test		MR-Egger		MR-PRESSO global test *p* value
		*Q* value	*p* value	Intercept	*p* value	
Television watching	OSA	125.304	0.076	−0.010	0.146	0.102
Computer use	OSA	90.651	0.092	0.0002	0.979	0.071
Driving	OSA	6.264	0.180	−0.112	0.145	0.226
MVPA	OSA	13.932	0.604	0.011	0.486	0.595
VPA	OSA	12.296	0.056	0.016	0.800	0.070
OSA	Television watching	3.248	0.197	−0.004	0.865	NA
OSA	Computer use	7.645	0.105	0.008	0.392	0.168
OSA	Driving	5.018	0.285	0.005	0.556	0.366
OSA	MVPA	3.605	0.307	−0.010	0.349	0.404
OSA	VPA	4.034	0.401	−0.003	0.498	0.355

OSA, obstructive sleep apnea; MVPA, self-reported moderate-to-vigorous physical activity; VPA, self-reported vigorous physical activity.

In the reverse MR analyses exploring the causal associations between OSA and computer use, driving, MVPA, and VPA, all *p*-values from the Cochran’s Q test, the MR-Egger intercept test (Supplementary Figures S9–S12), and the MR-PRESSO global test were > 0.05 (Table 2). This indicates no significant heterogeneity or horizontal pleiotropy in these analyses. However, the situation was different when investigating the effect of OSA on television watching. The Cochran’s Q test showed heterogeneity (*p* = 1.94 × 10^−4^), and the MR-PRESSO analyses showed that rs10928560 and rs4837016 were sources of bias (Supplementary Table S7). After removing these two SNPs, we re-performed MR analyses and sensitivity tests on the relationship between OSA and television watching (Figure 4; Table 2). Due to the reduction to only three remaining SNPs, conducting the MR-PRESSO test was not feasible. To solve this problem, we appropriately lowered the genome-wide significance threshold to *p* < 5 × 10^−7^, thereby expanding the number of available SNPs while keeping all other screening conditions constant (Supplementary Table S8). This strategy allowed us to include 8 SNPs for a complete sensitivity analysis. Re-analyses after removing outliers (Supplementary Table S9) similarly showed that OSA did not causally affect television watching (Supplementary Figure S13). In addition, all *p*-values for the Cochran’s Q test, MR-Egger intercept test (Supplementary Figures S14, S15), and MR-PRESSO global test were > 0.05, confirming no significant horizontal pleiotropy or heterogeneity in the analyses (Supplementary Table S10). Supplementary Figures S16–S21 display the LOO analyses.

## 4 Discussion

According to current knowledge, there is still no complete cure for OSA. Continuous positive airway pressure (CPAP) therapy is the most widely used clinical approach (4). However, low patient compliance rates significantly hamper the effectiveness of CPAP therapy. Furthermore, once CPAP therapy is discontinued, OSA symptoms rapidly recur (47). Despite the absence of a complete cure for OSA, adopting proactive preventive measures can mitigate the likelihood of its occurrence. Therefore, acquiring a more profound understanding of the risks and protective factors associated with OSA is imperative. Previous studies have identified several modifiable risk factors as critical targets for the prevention and treatment of OSA, including overall health rating, napping during the day, BMI, body fat mass, body water mass, hypertension, and education (27). Building on these findings, our study further explored the causal relationship between LSB/PA and OSA. We found that leisurely television watching and non-work-related computer use are risk factors for OSA, while VPA may be a protective factor. However, our findings do not support that driving and MVPA causally affect OSA risk.

The present study found that sedentary behavior increases the risk of OSA, aligning with findings from multiple prior observational studies (13–15). Unobstructed venous return is fundamental to maintaining good health. However, persistent sedentary behavior can impede venous return, accumulating fluid in the distal lower extremities’ intravascular and interstitial spaces (1). This accumulated fluid moves toward the neck when lying down to sleep at night, increasing upper airway resistance and contributing to the development of OSA (48). Additionally, a meta-analysis has indicated that sedentary behaviors contribute to obesity (49), which in turn increases the accumulation area and volume of fat deposits in the posterior wall of the maxilla, the pharynx, and the upper airways (50). During nighttime sleep, this accumulation can obstruct the upper respiratory tract, triggering OSA (50). Interestingly, among the three LSB phenotypes, we did not find significant evidence of causality between driving and OSA. The impact of different LSB on OSA risk may vary. In contrast to driving, watching television and using the computer are sedentary behaviors that involve screen exposure (SE-SB). The meta-analysis revealed that SE-SB is linked to an increased risk of depression (51). Depression is often accompanied by increased levels of pro-inflammatory cytokines, which can cause neurological damage and disrupt normal circadian rhythms, increasing the risk of OSA (52, 53). A recently published MR study confirmed the causal relationship between depression and OSA (54). Additionally, watching television and using the computer are more likely to occur later in the day, closer to bedtime. This behavior can lead to sleep deprivation and circadian misalignment, which in turn can exacerbate metabolic dysfunction and inflammation, ultimately influencing the occurrence of OSA (14).

Exercise is recognized as the second most effective treatment for OSA after CPAP (55). Although the mechanisms by which exercise improves OSA are not yet fully understood, several improvement pathways have been suggested. These include reduced fluid accumulation in the neck (56, 57), improved sleep quality (58), increased upper airway dilator muscle tension (59), and fat redistribution (3). It has also been suggested that different phenotypes of PA, such as exercise intensity, may affect OSA risk differently. The available studies have not reached consistent conclusions regarding the relationship between different PA intensities and OSA (16, 60, 61). Therefore, we conducted an MR analysis to evaluate the causal relationship. The results of this MR study suggest that the protective effect of PA against OSA is limited to VPA, while MVPA did not significantly reduce the risk of OSA. This finding indicates that light weight-bearing exercise and slow cycling may not significantly protect against OSA. It is important to recognize that previous studies have primarily used observational designs, which could not completely eliminate confounding factors or reverse causality interferences, potentially leading to different findings. Our analysis showed a causal relationship between VPA and OSA, which may be based on several mechanisms. Firstly, VPA improves slow-wave sleep quality by increasing slow-wave stability (62). Improving slow-wave sleep quality can lead to an improvement in OSA (59). Secondly, VPA can redistribute body fat, reducing the risk of OSA by decreasing fat accumulation around the neck and improving upper airway patency (63). Exercise is recognized as the second most effective treatment for OSA after CPAP (52). Although the mechanisms by which exercise improves OSA are not yet fully understood, several improvement pathways have been suggested. These include reduced fluid accumulation in the neck (53, 54), improved sleep quality (55), increased upper airway dilator muscle tension (56), and fat redistribution (3). It has also been suggested that different phenotypes of PA, such as exercise intensity, may affect OSA risk differently. The available studies have not reached consistent conclusions regarding the relationship between different PA intensities and OSA (13, 57, 58). Therefore, we conducted an MR analysis to evaluate the causal relationship. The results of this MR study suggest that the protective effect of PA against OSA is limited to VPA, while MVPA did not significantly reduce the risk of OSA. This finding indicates that light weight-bearing exercise and slow cycling may not significantly protect against OSA. It is important to recognize that previous studies have primarily used observational designs, which could not completely eliminate confounding factors or reverse causality interferences, potentially leading to different findings. Our analysis showed a causal relationship between VPA and OSA, which may be based on several mechanisms. Firstly, VPA improves slow-wave sleep quality by increasing slow-wave stability (59). Improving slow-wave sleep quality can lead to an improvement in OSA (56). Secondly, VPA can redistribute body fat, reducing the risk of OSA by decreasing fat accumulation around the neck and improving upper airway patency (60). Although both high-intensity interval training (HIIT) and moderate-intensity continuous training (MICT) are effective in reducing body fat (64), HIIT is more effective in promoting fat redistribution in specific populations, such as postmenopausal women (65). HIIT can reduce neck circumference, which may reflect a reduction in fat accumulation in this area, thereby improving upper airway patency. This may explain why the present study found that only VPA has a protective effect against OSA. Thirdly, VPA may improve OSA by enhancing lower limb muscle function and reducing leg fluid retention. A recent study indicates that HIIT significantly improves muscle mass and lower limb muscle function in sedentary individuals, whereas MICT does not yield the same effects (66). This suggests that only VPA may significantly enhance lower limb muscle function, thereby improving OSA. Consequently, the reduced transfer of fluid from the legs to the airway during sleep leads to the expansion of the upper airway. Finally, individuals who engage in VPA may be more likely to follow a healthier diet and be less sedentary for extended periods (67).

Although several studies have suggested that OSA may lead to a reduction in PA (19, 20), our findings indicate that only VPA causally reduces the risk of OSA. This suggests that the association between OSA and PA may be unidirectional, and previous observational studies may have produced false-positive results.

Our study has several significant advantages. Firstly, compared to traditional observational studies, we utilized a bidirectional two-sample MR analysis to minimize the effects of confounders and reverse causality, providing more reliable causality estimates. Secondly, we selected IVW as our primary analysis method due to its higher statistical power compared to other MR analysis methods. However, IVW is susceptible to horizontal pleiotropy. To address this issue, we used the MR-PRESSO global test and the MR-Egger intercept test to ensure that SNPs were free of potential pleiotropy and to guarantee the robustness of our analytical results. Additionally, we minimized the type I error probability by obtaining exposure and outcome data from different cohorts to avoid overlap between samples.

While our study offers new perspectives into the potential causal relationships between LSB/PA and the risk of OSA, we must acknowledge several limitations. Firstly, since all GWAS data were collected from participants with European ancestry, it is important to note that the findings of this study may not be directly generalizable to other ethnic populations. Genetic associations can vary across different populations due to differences in allele frequencies, environmental interactions, and socioeconomic factors. Therefore, further research involving diverse ethnic groups is essential for a more comprehensive understanding of the genetic underpinnings and their potential clinical implications. Secondly, although we investigated the potential causal relationship between LSB/PA and OSA risk, further research is needed to elucidate the underlying mechanisms. Thirdly, our study used a self-reported questionnaire to collect data on LSB and PA. While self-reported questionnaires are cost-effective and suitable for large-scale population-based studies, they are subject to information bias, such as recall bias and social desirability bias. These biases can affect the accuracy and reliability of the reported data (68). Despite these limitations, self-reported questionnaires can directly reflect respondents’ experiences and remain a widely used data collection instrument due to their practicality and relatively high reliability (69). However, future studies should consider incorporating objective measures, such as accelerometers, to obtain more accurate assessments of LSB and PA. Fourthly, although MR helps to reduce confounding and reverse causation, there is still a potential for pleiotropy, where genetic variants influence the outcome through pathways other than the exposure of interest. We employed several sensitivity analyses, including MR-Egger regression, MR-PRESSO, and LOO analysis, to detect and adjust for pleiotropic effects. Nevertheless, these methods may not entirely eliminate pleiotropic bias. Finally, due to the use of GWAS pooled data and the lack of individual measurement raw data, we could not conduct analyses for specific subgroups, such as age and gender. Further studies with access to individual-level data are needed to explore these subgroup-specific effects.

## 5 Conclusion

In conclusion, our findings suggest that leisurely watching television and computer use are risk factors for OSA, while VPA may be a protective factor. Additionally, OSA does not affect PA or LSB levels. We recommend reducing sedentary activities, particularly watching television and computer use, and prioritizing VPA to reduce the risk of OSA. However, given that our data are primarily from individuals of European ancestry, further research is needed in diverse populations and settings to validate these findings and understand their broader applicability.

## Data availability statement

Publicly available datasets were analyzed in this study. This data can be found here: https://gwas.mrcieu.ac.uk.

## Ethics statement

The studies involving humans were approved by the North West Multi-centre Research Ethics Committee, The Coordinating Ethics Committee of the Hospital District of Helsinki and Uusimaa. The studies were conducted in accordance with the local legislation and institutional requirements. The human samples used in this study were acquired from another research group. Written informed consent for participation was not required from the participants or the participants’ legal guardians/next of kin in accordance with the national legislation and institutional requirements.

## Author contributions

HT: Conceptualization, Data curation, Formal analysis, Methodology, Visualization, Writing – original draft, Writing – review & editing. AW: Conceptualization, Validation, Writing – original draft, Writing – review & editing. HW: Data curation, Formal analysis, Visualization, Writing – review & editing. CZ: Conceptualization, Validation, Writing – review & editing. ZZ: Conceptualization, Project administration, Supervision, Writing – review & editing. JW: Conceptualization, Supervision, Validation, Writing – review & editing.

## Footnotes

^1^ https://gwas.mrcieu.ac.uk

^2^ https://www.finngen.fi/en

^3^ http://www.phenoscanner.medschl.cam.ac.uk/
