# Supplementary material for: The causality between leisure sedentary behaviors, physical activity and obstructive sleep apnea: a bidirectional Mendelian randomization study
Source: Front Public Health. 2024 Jun 21;12:1425060. doi: 10.3389/fpubh.2024.1425060 (PMC11224541; doi:10.3389/fpubh.2024.1425060)
Supplement: Supplementary file 2 [file Image_1.PDF]

# *Supplementary Figures*

Supplementary Figure S1  MR estimates results of causal effect of OSA on MVPA.

Supplementary Figure S2 Scatter plots for driving on OSA.

Supplementary Figure S3 Scatter plots for MVPA on OSA.

Supplementary Figure S4 Leave-one-out analysis for watching television on OSA.

Supplementary Figure S5 Leave-one-out analysis for computer use on OSA.

Supplementary Figure S6 Leave-one-out analysis for VPA on OSA.

Supplementary Figure S7 Leave-one-out analysis for driving on OSA.

Supplementary Figure S8 Leave-one-out analysis for MVPA on OSA.

Supplementary Figure S9 Scatter plots for OSA on using computer.

Supplementary Figure S10 Scatter plots for OSA on driving.

Supplementary Figure S11 Scatter plots for OSA on MVPA.

Supplementary Figure S12 Scatter plots for OSA on VPA.

Supplementary Figure S13 MR estimates results of causal effect of OSA on television watching.

Supplementary Figure S14 Scatter plots for OSA on television watching ($P < 5 \times 10^{-8}$).

Supplementary Figure S15 Scatter plots for OSA on television watching ($P < 5 \times 10^{-7}$).

Supplementary Figure S16 Leave-one-out analysis for OSA on computer use.

Supplementary Figure S17 Leave-one-out analysis for OSA on driving.

Supplementary Figure S18 Leave-one-out analysis for OSA on MVPA.

Supplementary Figure S19 Leave-one-out analysis for OSA on television watching ($P < 5 \times 10^{-8}$).

Supplementary Figure S20 Leave-one-out analysis for OSA on television watching ($P < 5 \times 10^{-7}$).

Supplementary Figure S21 Leave-one-out analysis for OSA on VPA.

| Exposure | Outcome | Method | *P* value | OR (95%CI) |
|---|---|---|---|---|
| OSA | MVPA | | | |
| | | IVW | **0.048** | |
| | | Weighted median | 0.092 | |
| | | MR Egger | 0.945 | |

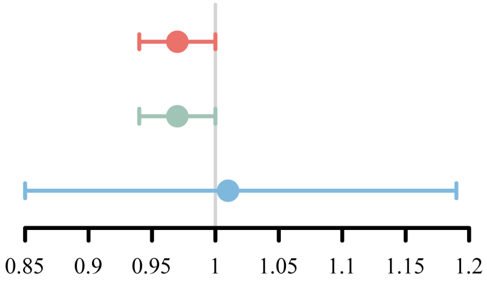


**Supplementary Figure S1. MR estimates results of causal effect of OSA on MVPA.** (OSA, obstructive sleep apnea; MVPA, self-reported moderate-to-vigorous physical activity; IVW, inverse variance weighted; OR, odds ratios; 95%CI, 95% confidence interval.)

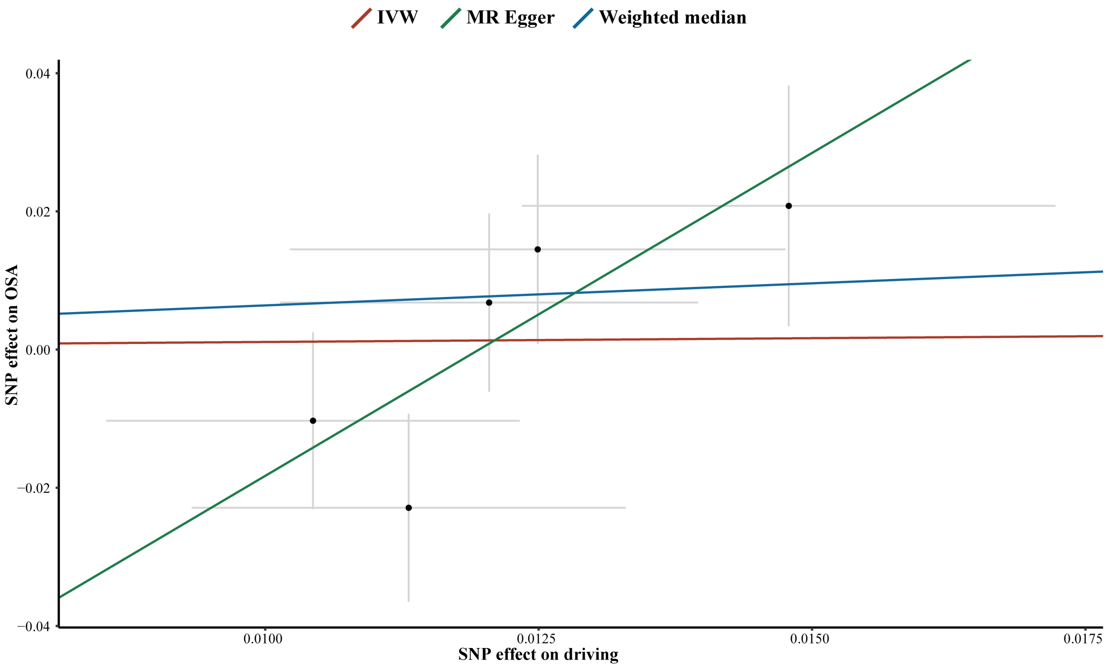


**Supplementary Figure S2. Scatter plots for driving on OSA.** (SNP, single-nucleotide polymorphism; OSA, obstructive sleep apnea; IVW, inverse variance weighted.)

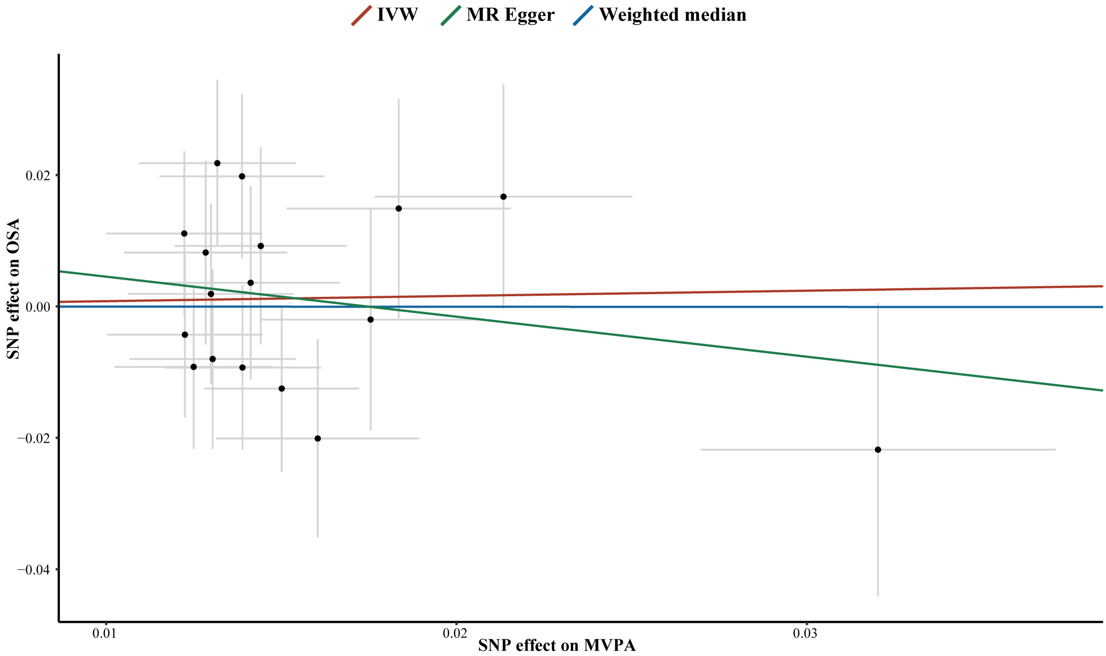


**Supplementary Figure S3. Scatter plots for MVPA on OSA.** (SNP, single-nucleotide polymorphism; OSA, obstructive sleep apnea; IVW, inverse variance weighted; MVPA, self-reported moderate-to-vigorous physical activity.)

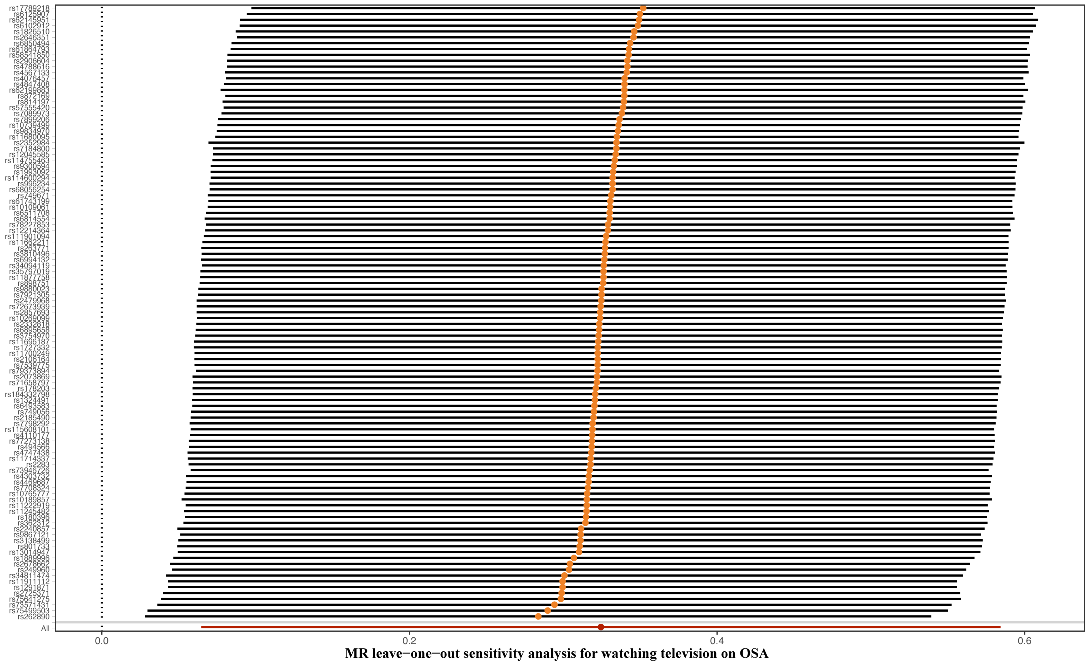


**Supplementary Figure S4. Leave-one-out analysis for watching television on OSA.**

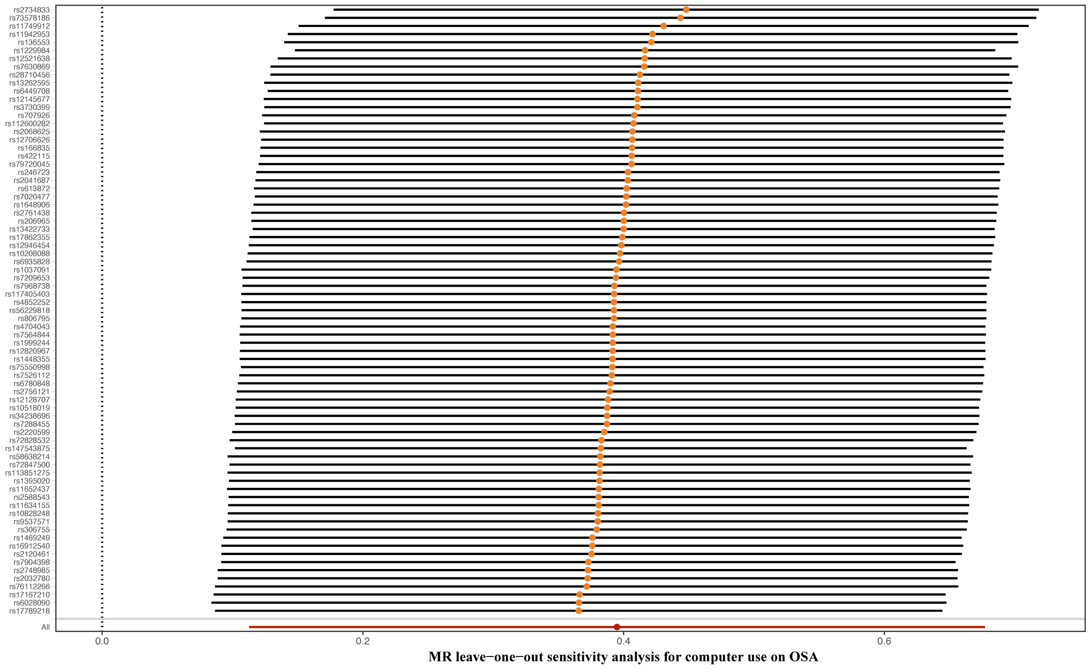


**Supplementary Figure S5. Leave-one-out analysis for computer use on OSA.**

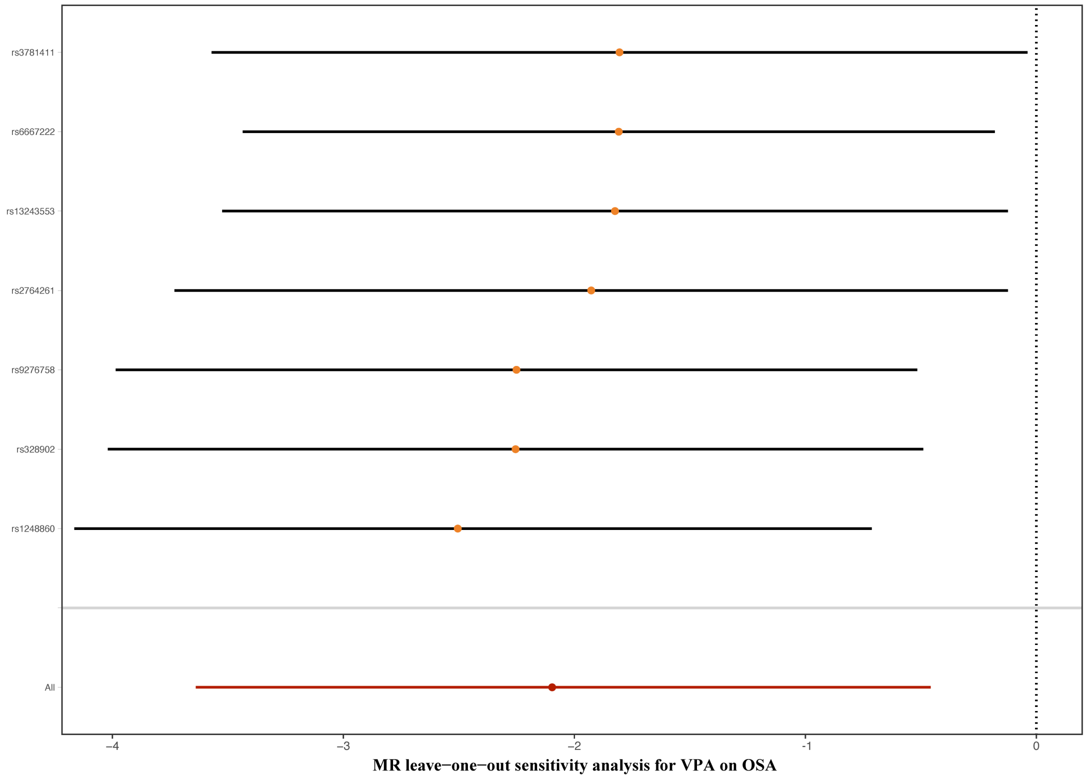


**Supplementary Figure S6. Leave-one-out analysis for VPA on OSA.**

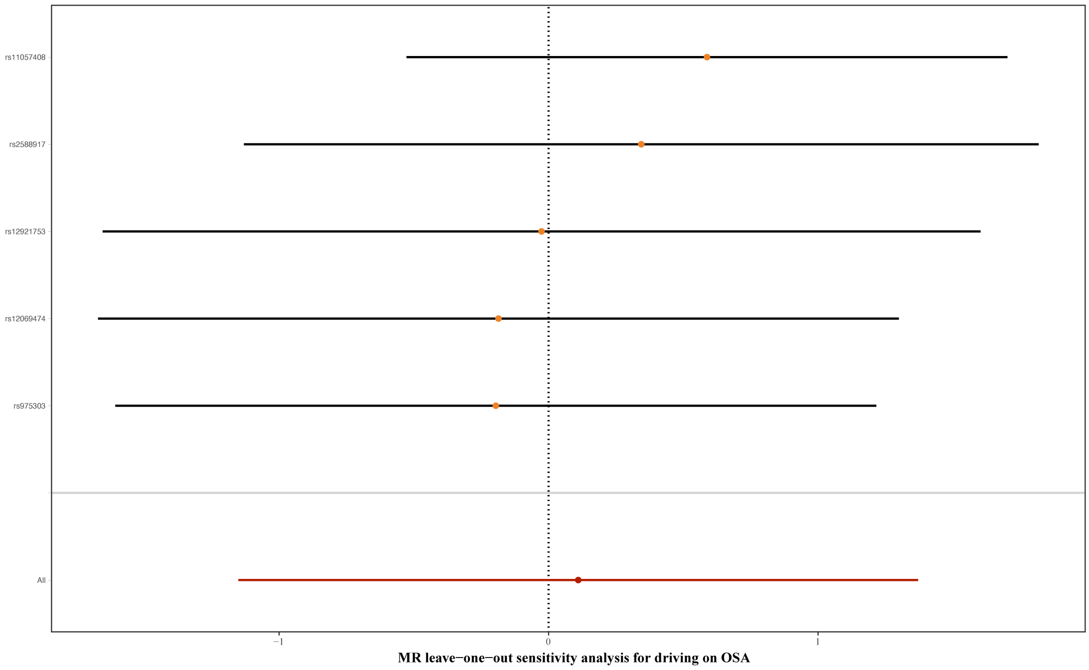


**Supplementary Figure S7. Leave-one-out analysis for driving on OSA.**

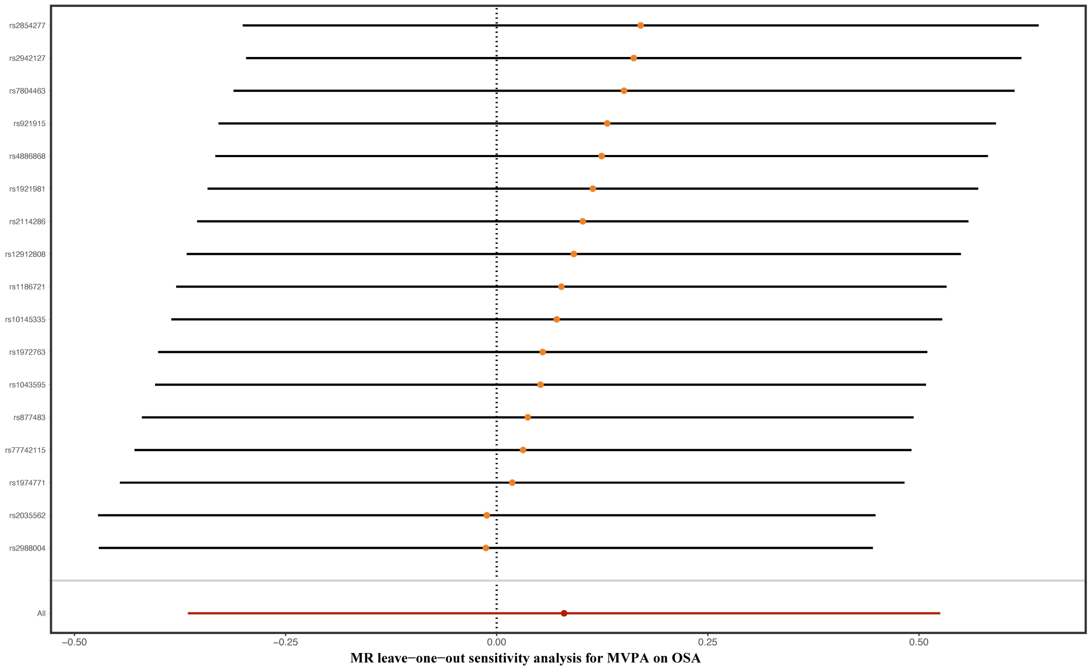


**Supplementary Figure S8. Leave-one-out analysis for MVPA on OSA.**

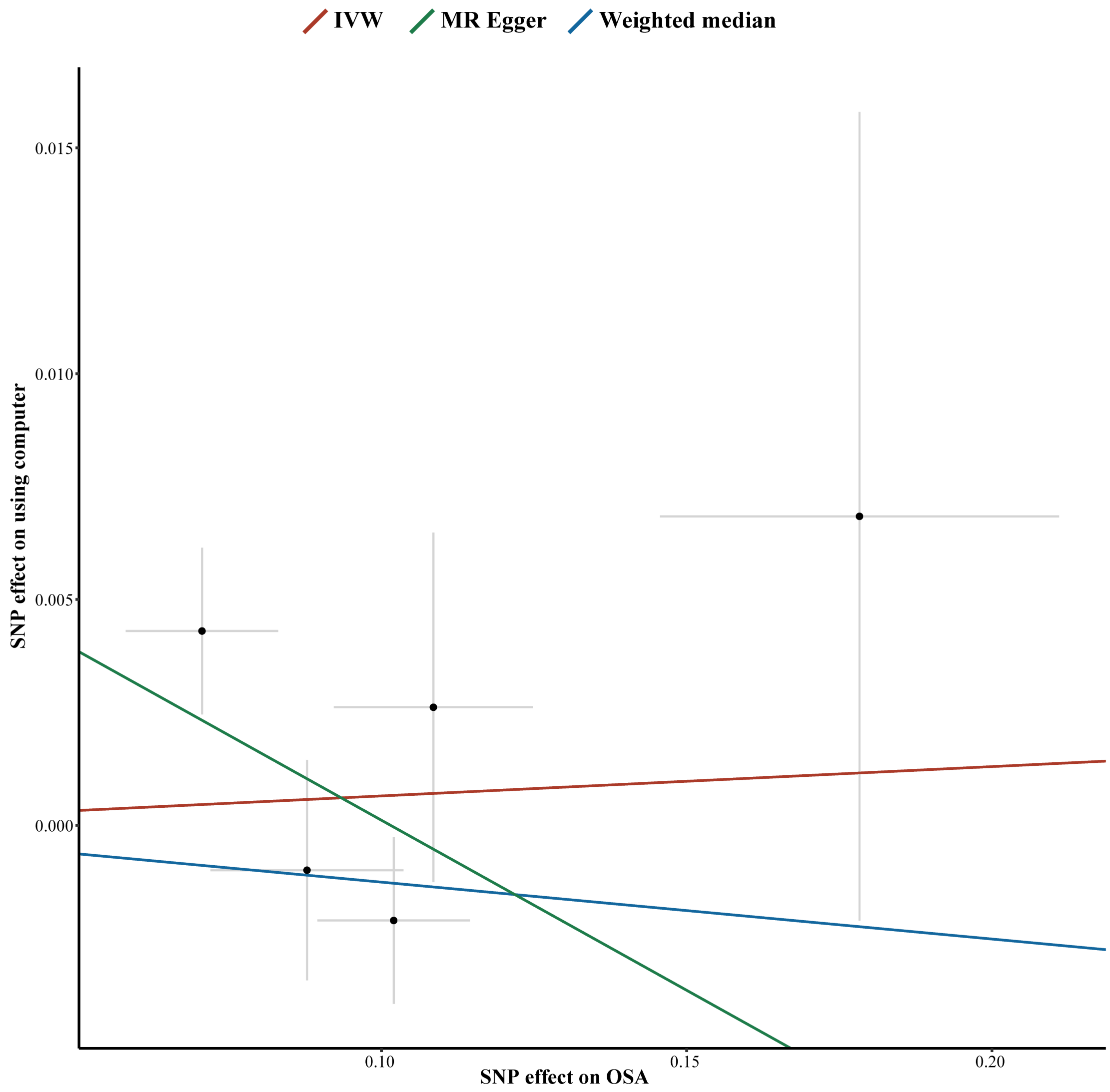


**Supplementary Figure S9. Scatter plots for OSA on using computer.** (SNP, single-nucleotide polymorphism; OSA, obstructive sleep apnea; IVW, inverse variance weighted.)

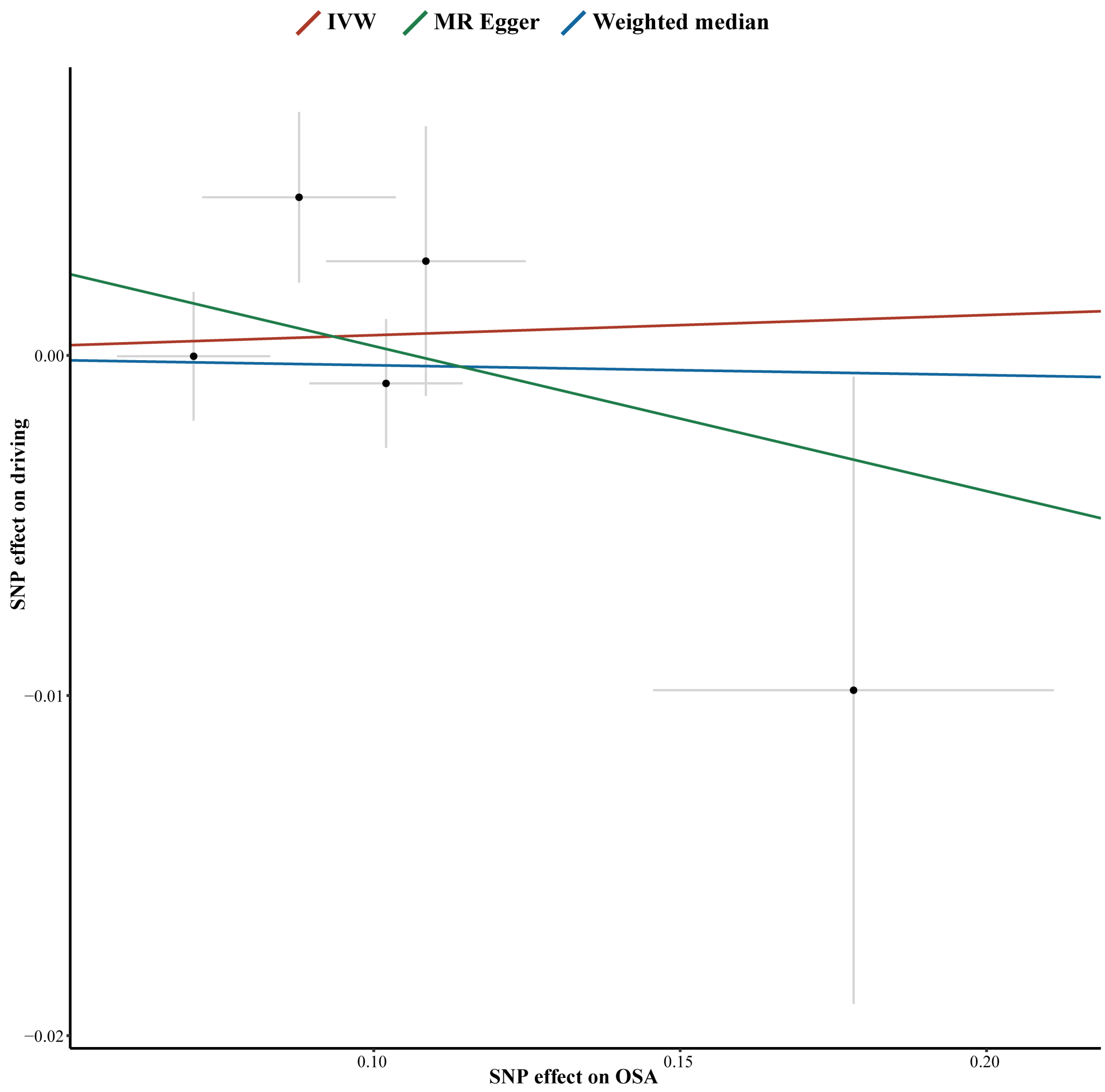


**Supplementary Figure S10. Scatter plots for OSA on driving.** (SNP, single-nucleotide polymorphism; OSA, obstructive sleep apnea; IVW, inverse variance weighted.)

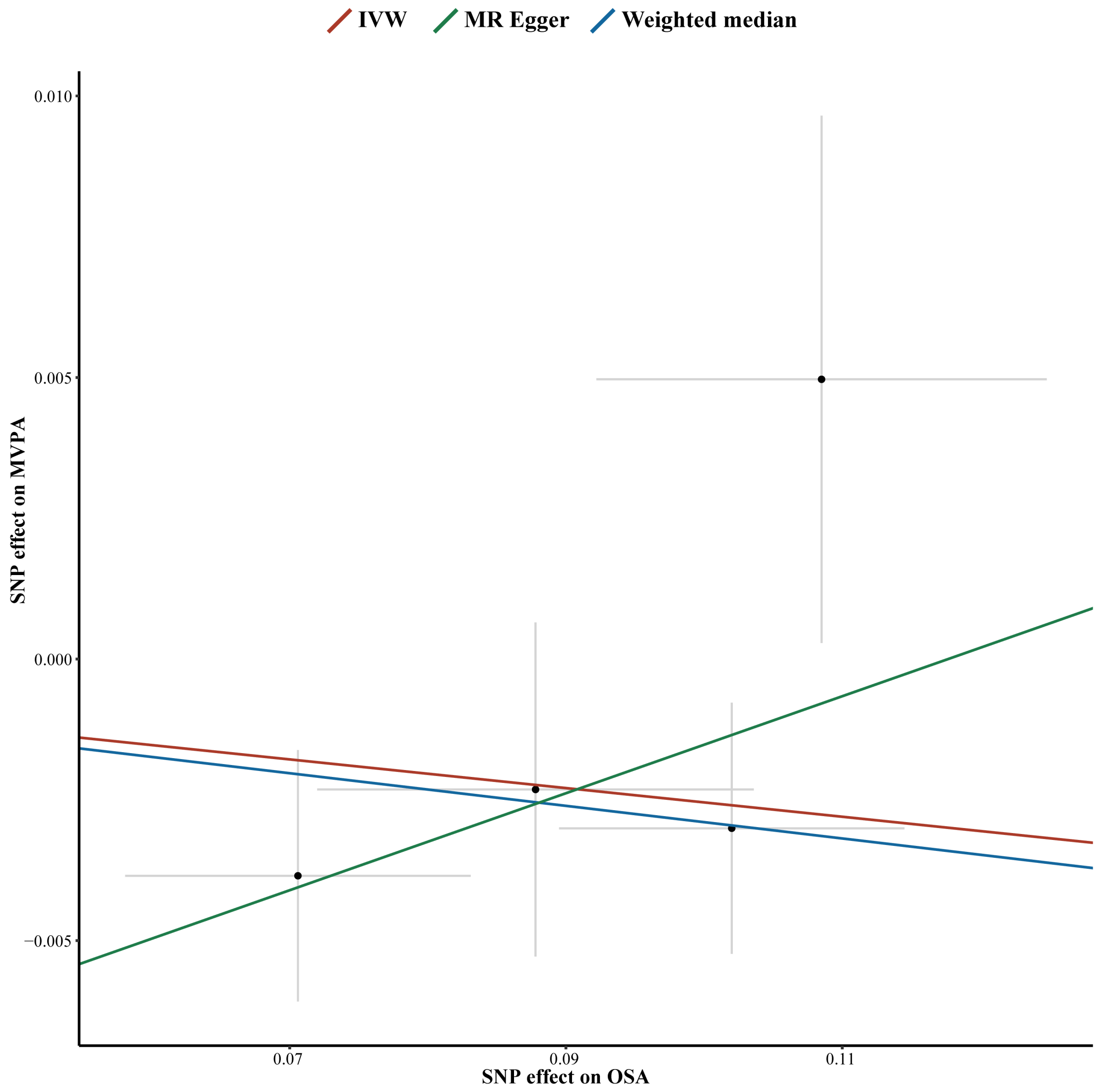


**Supplementary Figure S11. Scatter plots for OSA on MVPA.** (SNP, single-nucleotide polymorphism; OSA, obstructive sleep apnea; IVW, inverse variance weighted; MVPA, self-reported moderate-to-vigorous physical activity.)

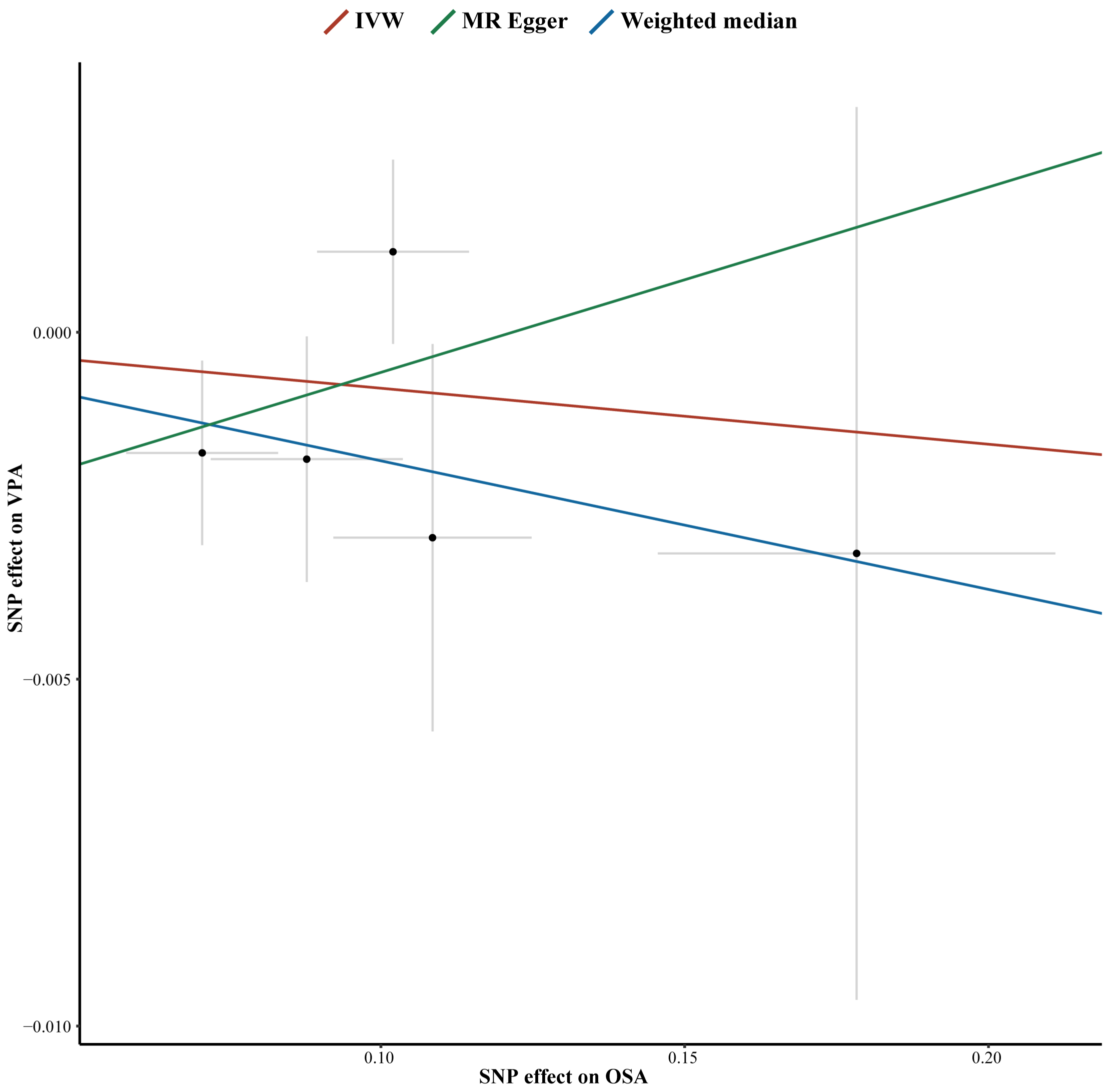


**Supplementary Figure S12. Scatter plots for OSA on VPA.** (SNP, single-nucleotide polymorphism; OSA, obstructive sleep apnea; IVW, inverse variance weighted; VPA, self-reported vigorous physical activity.)

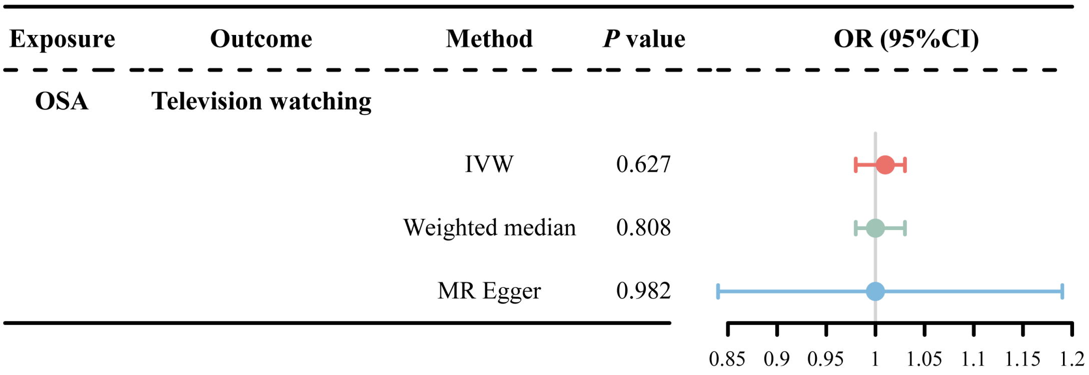


**Supplementary Figure S13. MR estimates results of causal effect of OSA on television watching.** (OSA, obstructive sleep apnea; IVW, inverse variance weighted; OR, odds ratios; 95%CI, 95% confidence interval.)

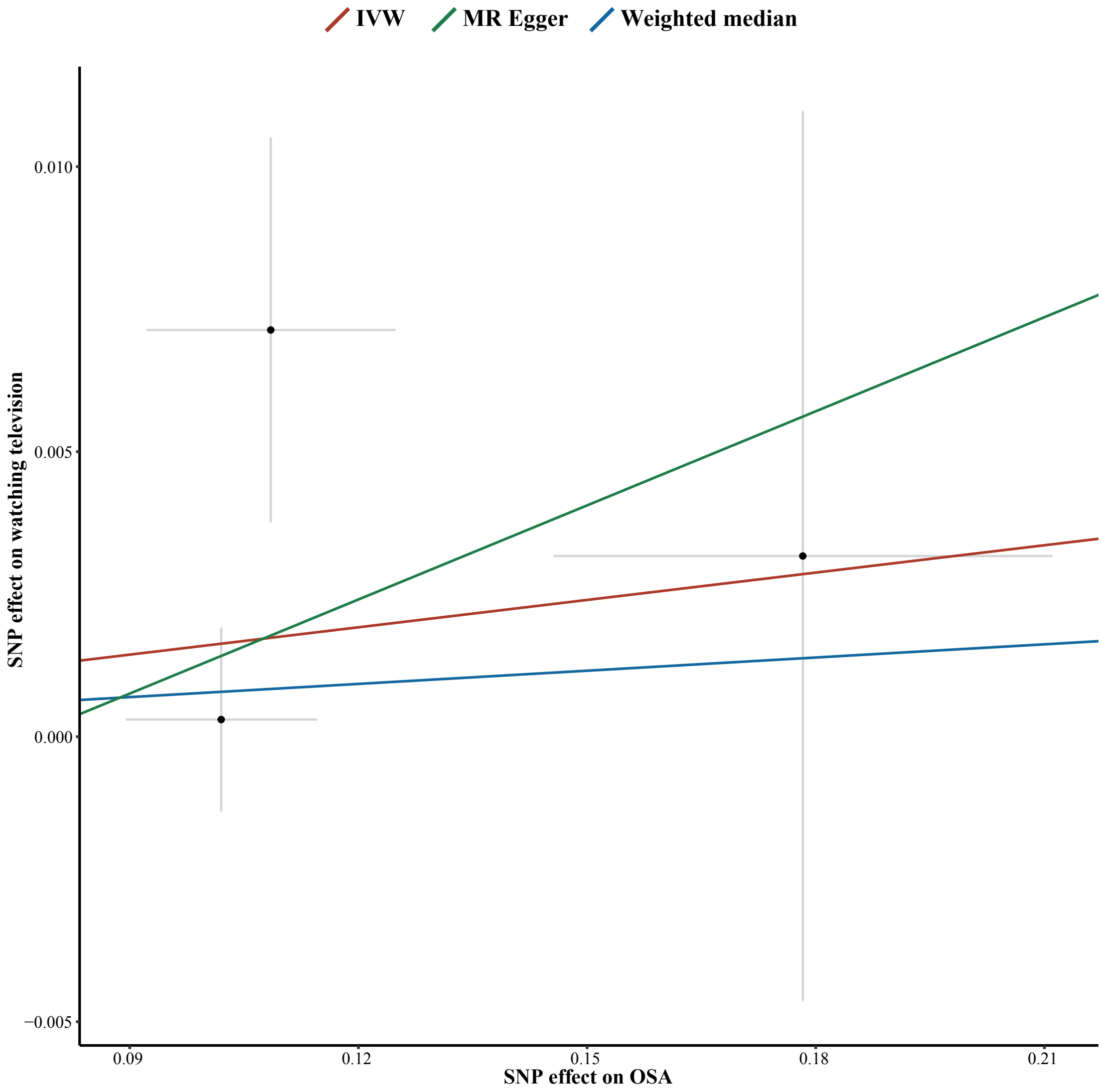


**Supplementary Figure S14. Scatter plots for OSA on television watching ($P < 5 \times 10^{-8}$).** (SNP, single-nucleotide polymorphism; OSA, obstructive sleep apnea; IVW, inverse variance weighted.)

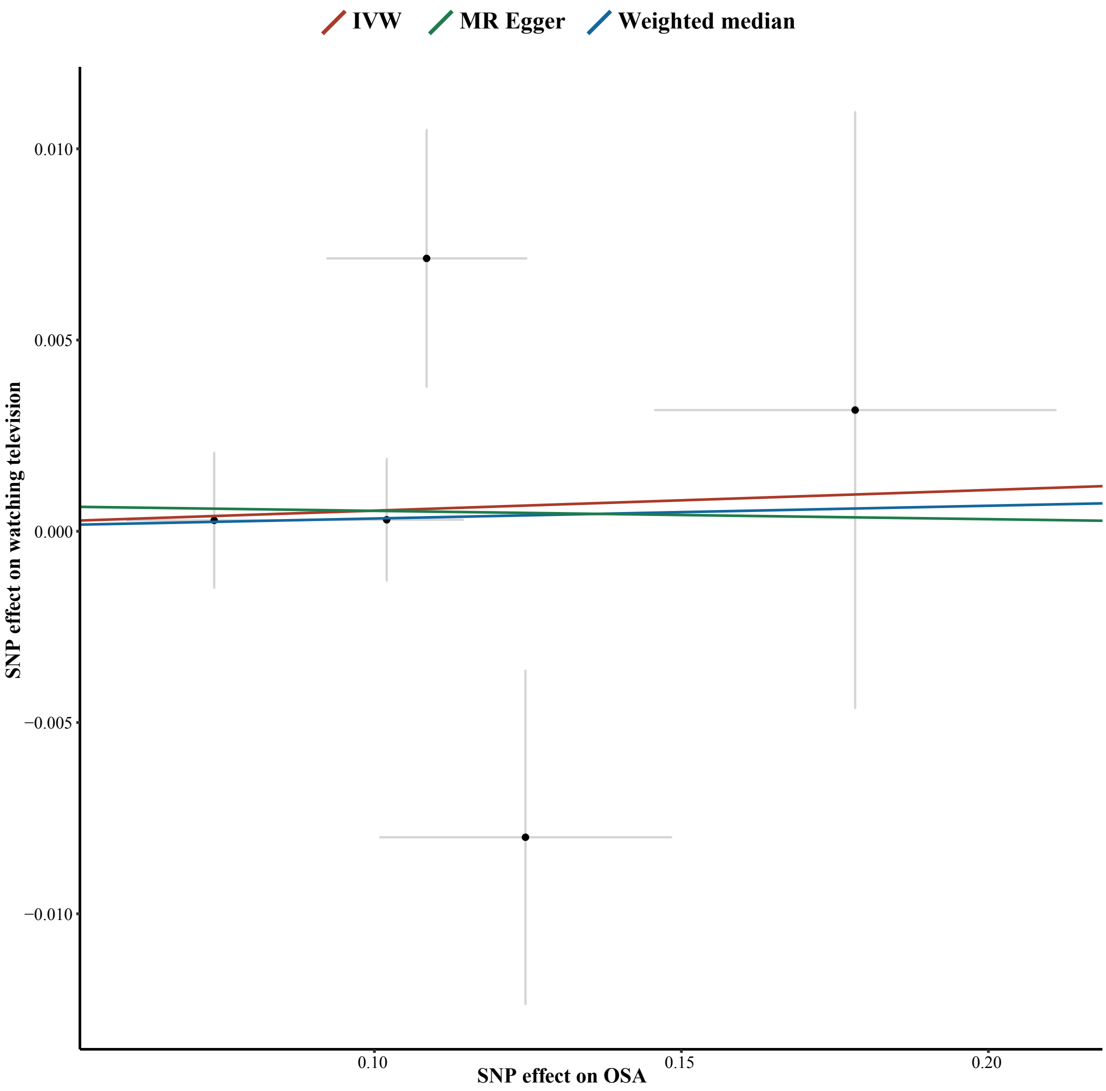


**Supplementary Figure S15. Scatter plots for OSA on television watching ($P < 5 \times 10^{-7}$).** (SNP, single-nucleotide polymorphism; OSA, obstructive sleep apnea; IVW, inverse variance weighted.)

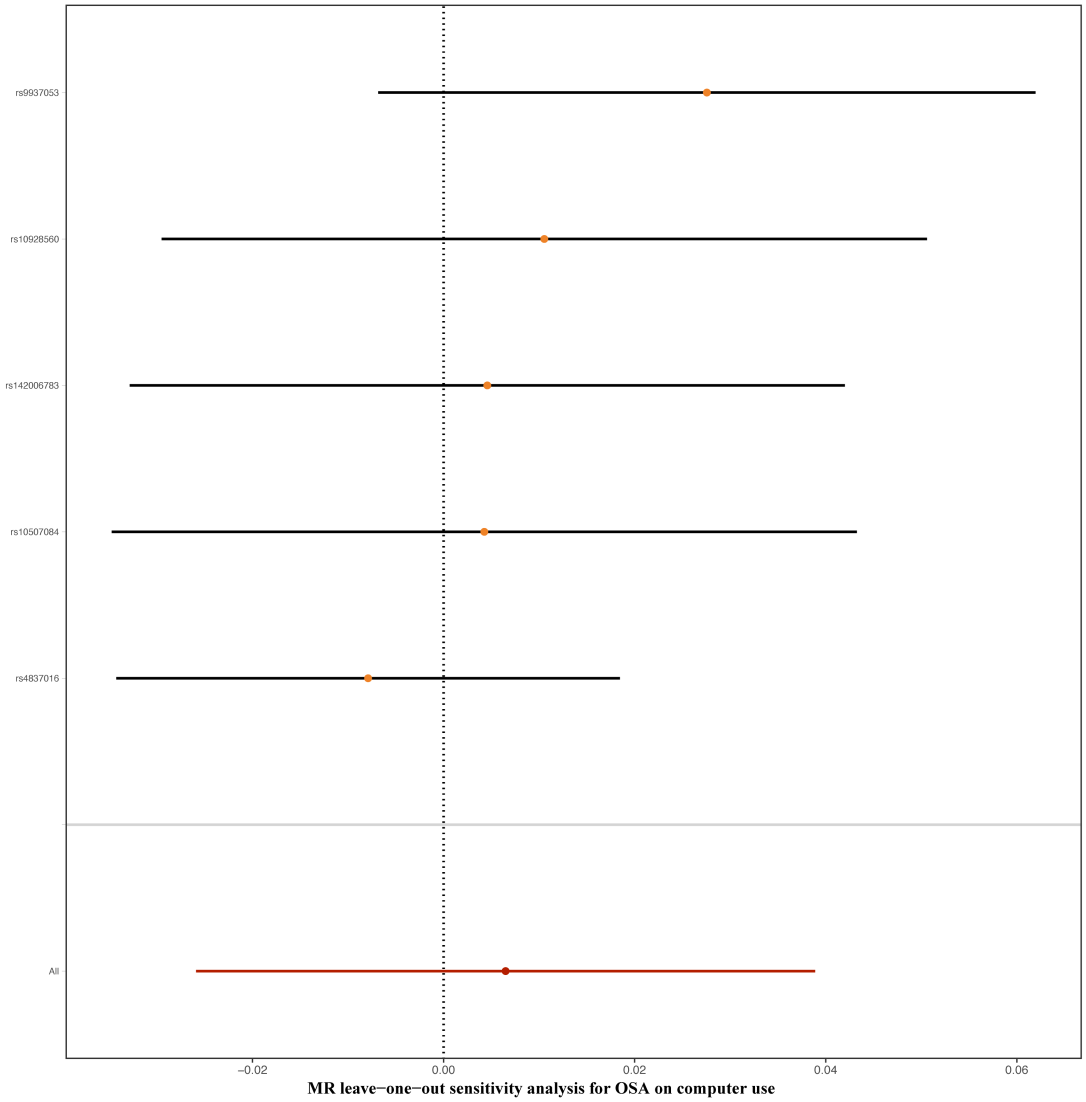


**Supplementary Figure S16. Leave-one-out analysis for OSA on computer use.**

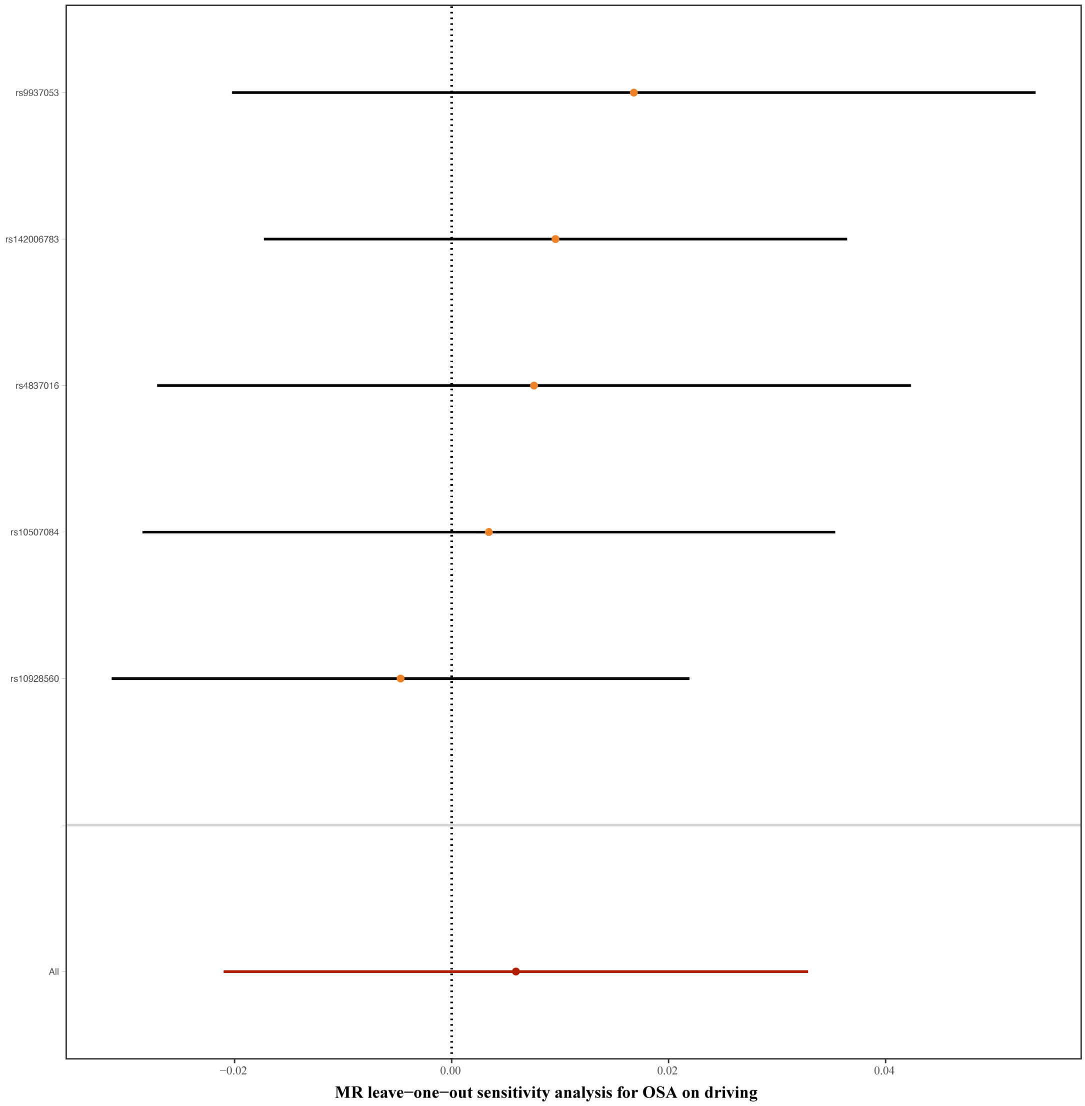


**Supplementary Figure S17. Leave-one-out analysis for OSA on driving.**

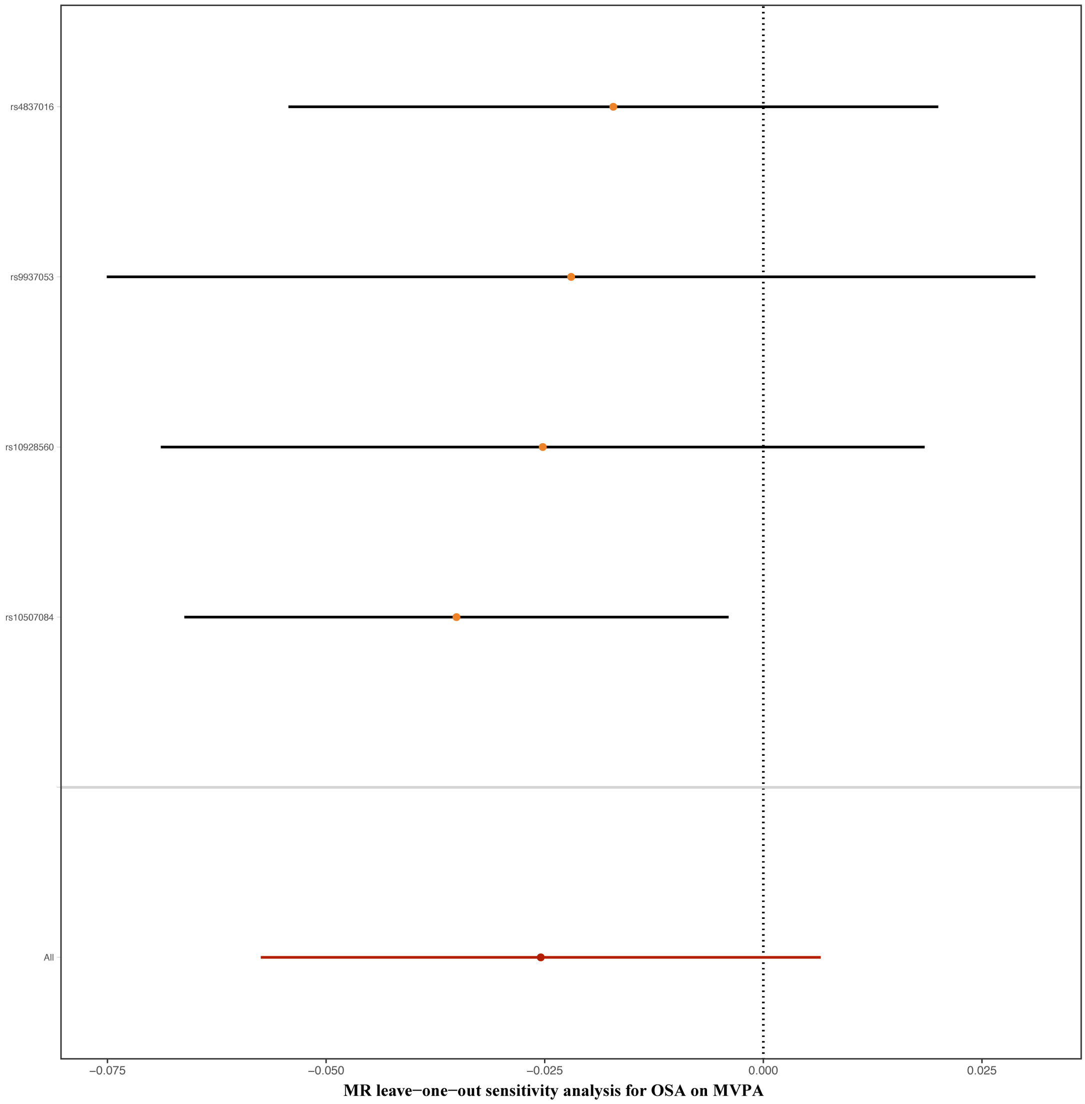


**Supplementary Figure S18. Leave-one-out analysis for OSA on MVPA.**

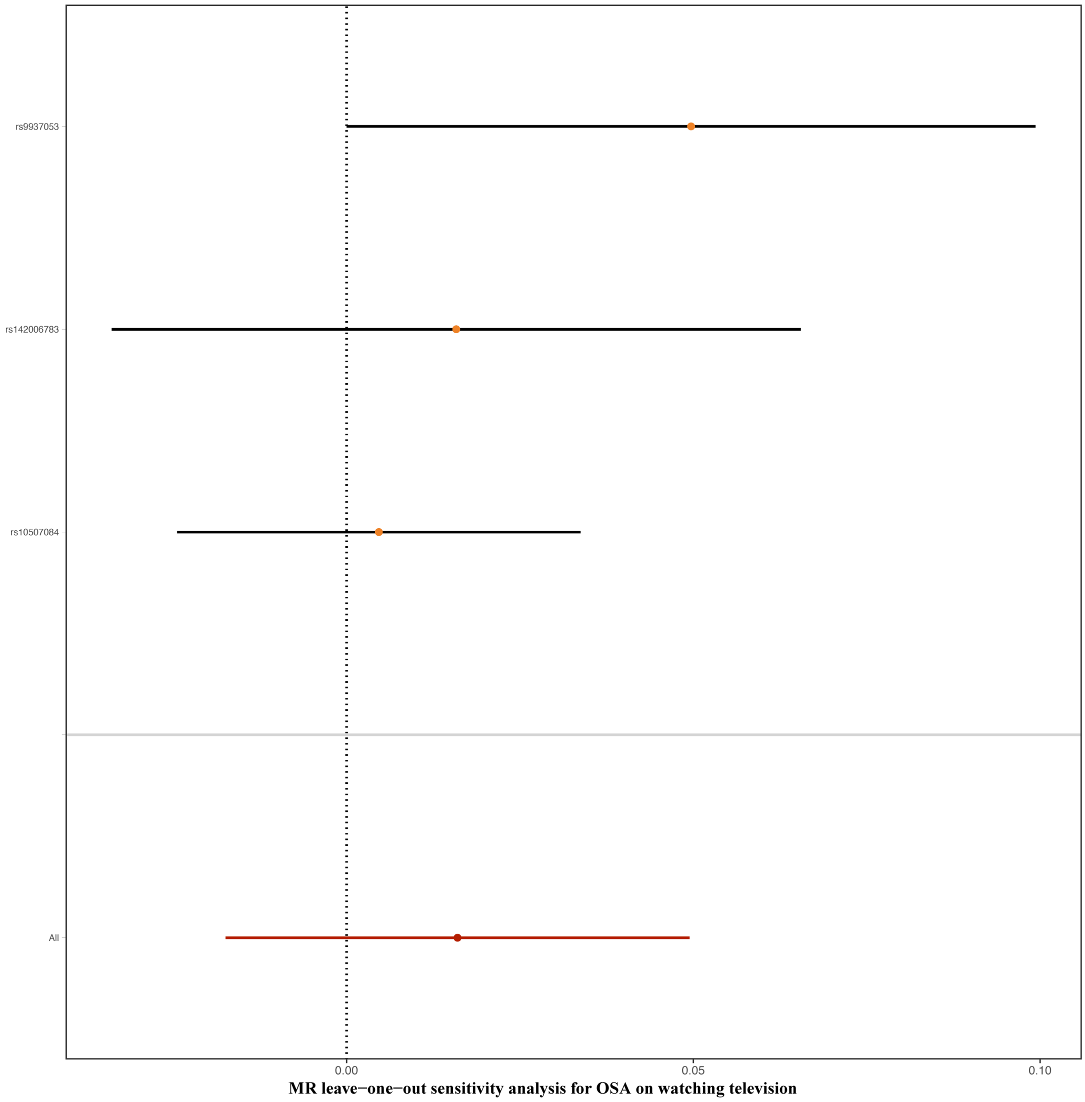


**Supplementary Figure S19. Leave-one-out analysis for OSA on television watching ($P < 5 \times 10^{-8}$).**

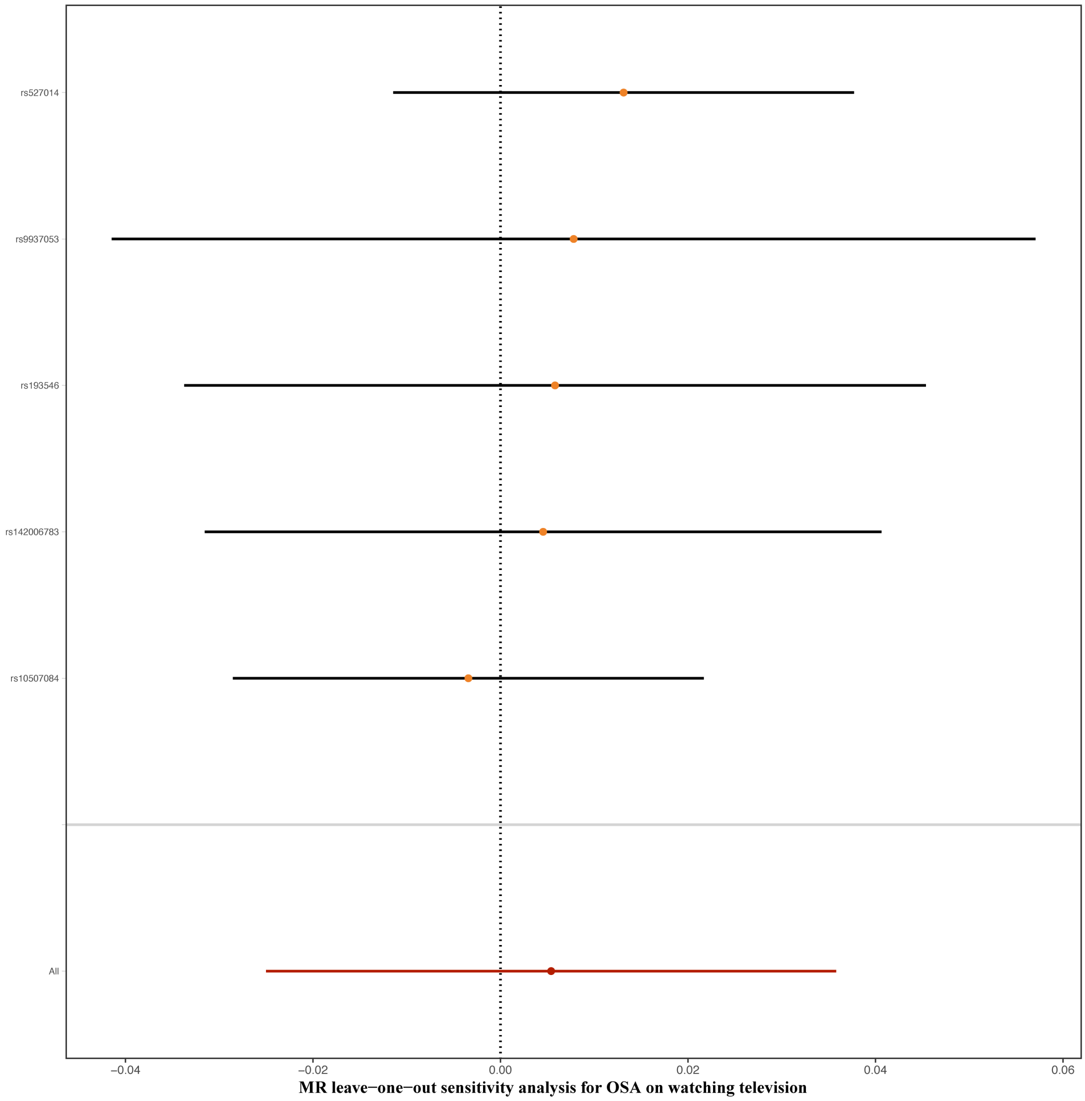


**Supplementary Figure S20. Leave-one-out analysis for OSA on television watching ($P < 5 \times 10^{-7}$).**

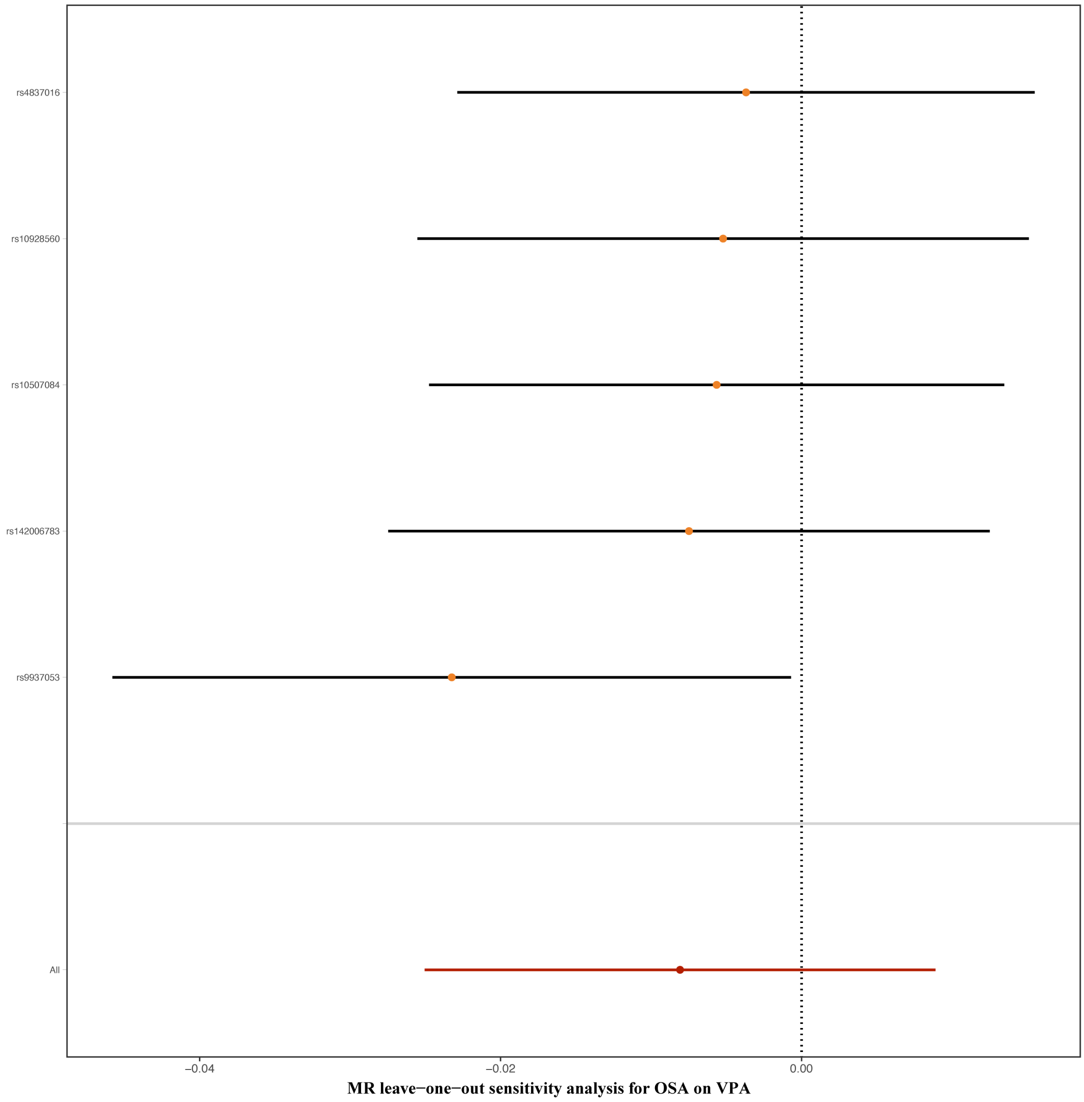


**Supplementary Figure S21. Leave-one-out analysis for OSA on VPA.**
